# Interface‐Confined Catalytic Synthesis of Anisotropic Covalent Organic Framework Nanofilm for Ultrafast Molecular Sieving

**DOI:** 10.1002/advs.202415520

**Published:** 2025-02-20

**Authors:** Jiahao Tang, Yu Liao, Zhenxiang Pan, Songjun Fang, Mingxiu Tang, Lu Shao, Gang Han

**Affiliations:** ^1^ College of Environmental Science and Engineering Tianjin Key Laboratory of Environmental Remediation and Pollution Control Nankai University 38 Tongyan Road Tianjin 300350 China; ^2^ MIIT Key Laboratory of Critical Materials Technology for New Energy Conversion and Storage State Key Laboratory of Urban Water Resource and Environment (SKLUWRE) School of Chemistry and Chemical Engineering Harbin Institute of Technology Harbin 150001 China

**Keywords:** confined catalysis, covalent organic framework, interfacial polymerization, molecular sieving, nanofiltration

## Abstract

Covalent organic frameworks (COFs) have emerged as prominent membrane materials for efficiently fractionating organic molecules and ions due to their unique pore structure. However, the fabrication of free‐standing COF nanofilms with high crystallinity remains an arduous undertaking, and feasible methods that can enable precise control over the film microstructure are barely reported. This work conceives an exquisite interface‐confined catalytic strategy to prepare Tp‐BD(OH)_2_ COF nanofilm with an anisotropic structure analogously to conventional polymeric membranes. Experimental data and molecular simulations reveal that the hydroxyl groups on the framework substantially capture and anchor the acid catalyst through hydrogen bonding interactions at the incipient stage of interfacial polycondensation, instigating confined catalysis and self‐termination reaction at the interface. The distinctive asymmetric structure endows the Tp‐BD(OH)_2_ COF nanofilm with a record‐breaking pure water permeance of 525.3 L m^−2^ h^−1^ bar^−1^ and unprecedented dye/salt selectivity of 648.6, surpassing other reported COF films and state‐of‐the‐art nanofiltration membranes, as well as enduring structural durability and chemical stability. The implemented interface‐confined catalysis strategy opens up a new avenue for regulating the COF nanofilm microstructure and holds broad prospects for the rational design of high‐performance membranes for sustainable water purification and treatment.

## Graphical abstract

This study develops an innovative interface‐confined catalytic synthesis strategy to molecularly construct anisotropic Tp‐BD(OH)₂ COF membranes with record‐breaking pure water permeance of 525.3 L m^−2^ h^−1^ bar^−1^ and exceptional molecule/ion selective factor up to 648.6, opening up a new avenue for prestigious nanostructure regulation of COF membranes for precise separations.

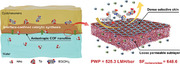

## 1 Introduction

Anthropogenic water pollution in tandem with the rapidly increased water demand necessitates an imminent use of untapped water abound in wastewater and seawater to alleviate water scarcity.^[^ ^1^ ^]^ The recent worldwide movement toward low‐carbon economic practices and sustainable development stimulates vigorous efforts in exploring energy‐efficient separation technologies.^[^ ^2^ ^]^ Membrane nanofiltration (NF), featuring a phase‐free conversion physical separation, has emerged as a vigorous separation technology for precisely differentiating organic molecules and salt ions pertinent to widespread applications in water reuse and chemical recovery due to its prominent advantages of high separation efficiency, low energy requirement, compact design, ease of operation, and environmentally benign characteristics.^[^ ^3^ ^]^ Fully exerting the advantages of NF in this arduous “fit‐for‐purpose” separation relies on the development of advanced semipermeable membranes with the appropriate microstructure in accordance with the size and charge differences between organic molecules and ions.

Polymeric membranes directly prepared by phase inversion and polyamide thin‐film composite membranes fabricated via interfacial polymerization are state‐of‐the‐art NF membranes that have achieved thrilling commercial successes.^[^ ^4^ ^]^ The widespread implementation of these membranes leans upon their asymmetric structure with an ultra‐thin selective skin and a support layer, affording high water permeation flux and robust mechanical durability. Nonetheless, they are markedly constrained by a deleterious trade‐off in the precise fractionation of organic molecules and ions, where membranes with high water permeance inevitably lack adequate selectivity.^[^ ^5^ ^]^ The prevailing membrane separation theories reveal that this ubiquitous, pernicious trade‐off is imposed by the intrinsic structure limitations of the polymer materials, where the stochastic chain packing poses challenges to precisely control the pore structure, yielding a broad distribution of pore sizes, tortuous pore channels, and low porosity, which are hard to circumvent by solely engineering the membrane structure.^[^ ^3^, ^6^ ^]^ The fundamental constraints of polymeric membranes motivate a proliferative discovery of alternative membrane materials that allow penetrants to transport by single‐file diffusion instead of stochastic partitioning through the membrane. Among the emerging porous materials, covalent organic frameworks (COFs) represent a class of porous organic crystalline materials with 2 or 3D structures formed through reactions between organic precursors in strong covalent bonds to afford exceptional material stability in a wide range of solvents and conditions.^[^ ^7^ ^]^ In the context of NF applications, the long‐range ordered straight‐through pore structure (pore size varying from 0.8 to 4.0 nm) and highly tunable chemical environment, high inherent porosity, and excellent stability render COFs an ideal class of membrane materials to facilitate the selective sieving of organic molecules and ions.^[^ ^7^, ^8^ ^]^ Nonetheless, COFs still experience many daunting limitations, particularly the inferior feasibility and practicality of preparing defect‐free COF membranes with adequate crystallinity. The inability to precisely regulate the microstructure and mass transfer behavior of COF membranes at the molecular level also poses an obvious obstacle to advancing their applications.

Prevalent approaches that have been recently adopted to prepare COF membranes include in situ growth,^[^ ^9^ ^]^ phase switching,^[^ ^10^ ^]^ vacuum‐assisted assembling,^[^ ^11^ ^]^ and interfacial polymerization (IP).^[^ ^12^ ^]^ The IP approach leverages the advantages of interface‐constrained step‐growth polymerization in which the precursors react at the interface between two immiscible phases, offering a promising platform for synthesizing continuous COF nanofilms with finely tuned crystallinity and emergent properties.^[^ ^7^, ^8^, ^13^ ^]^ Despite the progress, one‐step synthesis of highly crystalline IP‐COF membranes is still at an extremely rudimentary level owing to the solubility limitation of precursors and countless screenings of suitable reaction conditions by trial and error.^[^ ^14^ ^]^ Trailblazing studies demonstrate that extraordinary water permeance was barely achieved by the recently reported IP‐COF membranes, usually less than 200 L m^−2^ h^−1^ bar^−1^ (LMH bar^−1^), even though they possess relatively high crystallinity, which is contrary to the intended benefits of their vertically aligned through‐channels in facilitating water transport.^[^ ^12^, ^15^ ^]^ In addition to the randomly stacked crystal domains within the IP‐COF membrane and the presence of amorphous fragments,^[^ ^12^, ^16^ ^]^ their symmetric cross‐section macrostructure is responsible for the inferior water permeance, where a thick structure elongates the molecule transport pathways, thereby increasing transport resistance and diminishing water permeance,^[^ ^17^ ^]^ while a thin structure is imperative for enhancing mass transport but inevitably deteriorates the mechanical durability and possibly membrane selectivity.^[^ ^11^, ^18^ ^]^ Drawing inspiration from the asymmetric structure of conventional polymeric membranes, we believe that anisotropic IP‐COF membranes with a dense selective skin and a porous sublayer, a structure previously given limited attention, can address this dilemma, where the dual‐layer structure would significantly enhance the permeation of water without sacrificing membrane selectivity, holding great promise in overcoming the formidable permeance/selectivity trade‐off in precise nanofiltration. Unfortunately, efforts thus far have yet to converge on a common strategy to accomplish this arduous undertaking with a one‐step IP synthesis protocol.

**Figure 1:**
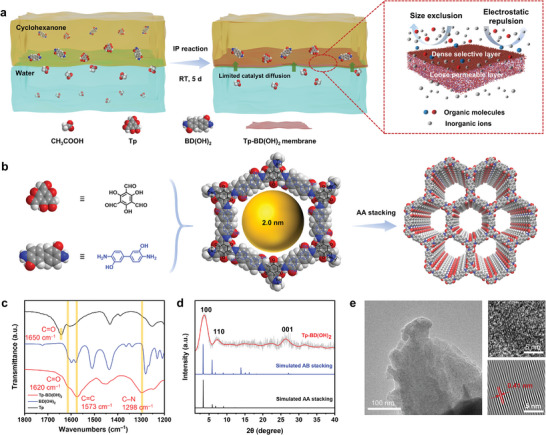
Synthesis, chemical structure, and characterization of the Tp‐BD(OH)_2_ COF membrane. a) Schematic illustration of the interface‐confined catalytic synthesis for molecular construction of anisotropic COF membrane with a dual‐layer asymmetric structure for selective fractionation of organic molecules and salt ions. b) Schematic diagram of the BD(OH)_2_ amine and Tp aldehyde precursors for COF synthesis, and the chemical skeleton and stacking mode of the obtained Tp‐BD(OH)_2_ COF membrane. c) FT‐IR spectra of Tp and BD(OH)_2_ precursors and the Tp‐BD(OH)_2_ COF membrane. Wavenumbers range from 1800 to 1200 cm^−1^. d) Experimental XRD patterns (gray curve: raw data; red curve: fitting curve), simulated AA stacking mode (black solid line), and simulated AB stacking mode (blue solid line) of the Tp‐BD(OH)_2_ COF membrane. e) HRTEM and the corresponding inverse fast Fourier transform (IFFT) images (bottom‐right) of the Tp‐BD(OH)_2_ COF membrane.

This study marks the first preparation of a self‐sustaining polycrystalline Tp‐BD(OH)_2_ COF membrane with a dual‐layer asymmetric microstructure via an innovative interface‐confined catalytic synthesis strategy (**Figure** **1**a). The dense selective skin with low thickness and exquisitely regulated size sieving and electrostatic repulsion selectivity endows the COF membrane with unprecedented permselectivity while the loose permeable sublayer provides rapid mass‐transfer channels with sufficient mechanical robustness. The obtained COF membrane breaks the trade‐off threshold in an exemplified application for textile water desalination, achieving a record‐breaking pure water permeance of 525.3 LMH bar^−1^ along with unprecedented dye/salt selectivity of as high as 648.6, far surpassing the performance of previously reported COF films and state‐of‐the‐art NF membranes. Advanced molecular simulations were employed to gain an in‐depth understanding of the underlying mechanism for the formation of the anisotropic COF membrane structure. This work provides a pivotal gateway to enrich our toolkit for the prestigious regulation of COF membrane microstructure and paves the way for developing ultra‐permeable and selective NF membranes for wide applications in sustainable water treatment and resource reclamation.

## 2 Results and Discussion

### 2.1 Synthesis and Structural Characterization of Anisotropic Tp‐BD(OH)_2_ COF Membrane

A self‐sustaining polycrystalline Tp‐BD(OH)_2_ COF membrane, for the first time reported in the literature, was synthesized under ambient conditions using an optimized interfacial polymerization (IP) approach (Figure 1a). During IP synthesis, the acetic acid (HAc) catalyst in the bottom water phase diffuses toward the upper cyclohexanone oil phase and then catalyzes the polycondensation of 1,3,5‐triformylphoroglucinol (Tp) and 3,3′‐dihydroxybenzidine (BD(OH)_2_) at the interface to form a free‐standing COF nanofilm. The Tp‐BD(OH)_2_ COF skeleton is molecularly constructed by forming imine covalent bonds through the co‐condensation of aromatic primary amines on BD(OH)_2_ and aldehyde on Tp in the presence of HAc, during which the lone pair of electrons of the amine N first attacks the activated carbonyl C to undergo nucleophilic addition, resulting in a hemiaminal intermediate, and then continues to eliminate a molecule of water, following by β‐ketoenamination (Figure 1b; Figures S1 and S2, Supporting Information).

Fourier‐transform infrared (FT‐IR) spectra (Figure 1c; Figure S3, Supporting Information) show the appearance of C─N and C═C characteristic peaks at 1298 and 1573 cm^−1^, respectively, which are attributed to the formation of ─C═C─N─, while the disappearance of peaks associated with ─NH_2_ (∼3200–3400 cm^−1^) and ─CHO (∼2891 and 1650 cm^−1^) in the precursors after reaction provides strong evidence for the formation Tp‐BD(OH)_2_ COF.^[^ ^11^, ^12^, ^17^ ^]^ Furthermore, the C═O peak at 1620 cm^−1^ confirms the presence of β‐ketoenamine bonds in the framework,^[^ ^19^ ^]^ while the wide peak at 3200–3600 cm^−1^ reveals that the ─OH on the BD(OH)_2_ precursor is retained.^[^ ^20^ ^]^ The X‐ray photoelectron spectroscopy (XPS) data in Figure S4 (Supporting Information) corroborates these results, where the semiquantitative analysis (Table S1, Supporting Information) and element distribution (Figure S5, Supporting Information) show that the COF membrane is composed of C, N, and O and the content of each element is close to the respective theoretical value of the Tp‐BD(OH)_2_ framework. The high‐resolution C 1s spectrum (Figure S4b, Supporting Information) is deconvoluted into four peaks of C─C/C═C (284.80 eV), C─O/C─N (286.13 eV), C═O (288.29 eV), and π‐π* excitation (291.92 eV),^[^ ^19^, ^20^, ^21^ ^]^ in good agreement with the Tp‐BD(OH)_2_ molecular structure. Moreover, the high‐resolution O 1s spectrum (Figure S4c, Supporting Information) shows two peaks at 531.19 and 533.26 eV, which are assigned to C═O and C─OH, respectively, underpinning the presence of ─OH and β‐ketoenamine bonds.^[^ ^10^, ^19^ ^]^ X‐ray diffraction (XRD) was then employed to access the polycrystalline structure of the Tp‐BD(OH)_2_ COF membrane, where prominent diffraction 2θ peaks at ∼3.6°, ∼6.4°, and ∼26.5° are observed in Figure 1d, which correspond to the (100), (110), and (001) reflections, respectively, well matching with the theoretical diffraction patterns of the corresponding COF topology and the literature data.^[^ ^19^, ^20^, ^22^ ^]^ The distinct sharp 2θ peak at ∼3.6° is ascribed to the reflection of the (100) plane, indicative of a highly ordered crystalline structure,^[^ ^19^ ^]^ while the broad peak at ∼26.5° is associated with the π−π stacking between COF layers. Notably, the experimental XRD patterns demonstrate that the Tp‐BD(OH)_2_ COF membrane has a stacking mode of AA type, resulting in highly ordered straight‐through channels with a pore opening of 2.0 nm in diameter. The detailed theoretical schematic diagrams of the stacking modes and the corresponding fractional atomic coordinates can be found in Figure S6 and Tables S2–S3 (Supporting Information). High‐resolution transmission electron microscopy (HRTEM) results reaffirm the well‐defined crystallinity and highly ordered porous structure of the COF membrane. As presented in Figure 1e, the membrane exhibits an evident thin lamellar structure with extremely ordered lattice fringes with large‐scale directional alignment (indicated by arrows) unequivocally seen on the surface. The lattice fringe spacing is determined to be 0.41 nm, which corresponds to the crystal plane of (250). All these results confirm the chemical features of the Tp‐BD(OH)_2_ skeleton that we intend to synthesis as well as the highly ordered porous structure and well‐defined crystallinity of the synthesized COF membrane.

**Figure 2:**
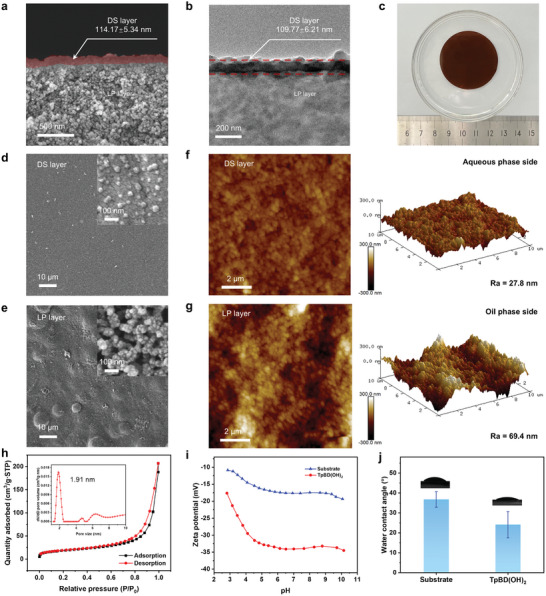
Morphological characteristics, microstructure, and physicochemical properties of the Tp‐BD(OH)_2_ COF membrane. a,b) Cross‐section a) FESEM and b) TEM images of the Tp‐BD(OH)_2_ COF membrane. The areas marked in red represent the dense selective (DS) layer. c) Digital photograph of the Tp‐BD(OH)_2_ COF membrane. The membrane shown here has a diameter of ≈4 cm, with a smooth and uniform surface. d–g) Surface FESEM and AFM images of the d,f) dense selective (DS) skin and e,g) loose permeable (LP) sublayer of the Tp‐BD(OH)_2_ COF membrane. The insets in the upper right corner of d,e) depict the microstructure of the surface under high magnification. h) N_2_ adsorption–desorption isotherms and the obtained pore size distribution of the Tp‐BD(OH)_2_ COF membrane stacked in AA mode. The sharp size distribution centered at 1.91 nm is consistent with the theoretical pore size of 2.0 nm. i) Zeta potential and j) Water contact angle of the substrate and the Tp‐BD(OH)_2_ COF membrane.

After initial chemical and crystalline structure confirmation, the morphological characteristics and microstructure of the Tp‐BD(OH)_2_ COF membrane were further characterized. Interestingly, a novel dual‐layer asymmetric structure consisting of a dense selective skin (denoted as the DS layer) and a loose permeable sublayer (denoted as the LP layer), analogously to the conventional polymeric membranes, is observed in the cross‐section of the COF membrane. As shown in the high‐resolution cross‐sectional field emission scanning electron microscopy (FESEM) (**Figure** **2**a; Figure S7a,b, Supporting Information) and transmission electron microscopy (TEM) images (Figure 2b), a uniform DS layer with a thickness of ≈114.17 ± 5.34 nm (measured from FESEM) and 109.77 ± 6.21 nm (measured form TEM) is formed on a highly porous LP sublayer. Compared to the LP sublayer, the DS layer exhibits a tight structure without any apparent microvoids. To the best of our knowledge, this is the first reported self‐sustaining Tp‐BD(OH)_2_ COF membrane with such an anisotropic structure. In the realm of water filtration, this unique structure could facilitate water permeation by shortening transport pathways but preserve the sieving ability of the COF structure, thereby affording a substantial increase in membrane permselectivity. The digital photograph shows that the Tp‐BD(OH)_2_ COF membrane has a visually smooth and pinhole‐free surface (Figure 2c), confirming the uniformity and integrity of the membrane at the macroscopic scale. Surface FESEM images (Figure 2d,e; Figure S7c,d, Supporting Information) and atomic force microscope (AFM) data (Figure 2f,g) further reveal that the membrane top surface on one side of the DS layer presents dense and smooth morphology with a low surface roughness (Ra = 27.8 nm), while the surface on the LP layer side becomes rough (Ra = 69.4 nm) and highly porous with macroscopic voids unequivocally seen on it. The significant differences in surface morphology of the two sides coincide with the asymmetric cross‐section structure of the Tp‐BD(OH)_2_ COF membrane.

N_2_ adsorption‐desorption isotherms were performed at 77 K to evaluate the specific surface area and pore size distribution of the Tp‐BD(OH)_2_ COF membrane. A relatively high Brunauer–Emmett–Teller surface area of 64.7 m^2^ g^−1^ concomitant with a sharp size distribution centered at 1.91 nm was obtained by the COF membrane (Figure 2h), which mirrors its theoretical pore size of 2.0 nm (Figure 1b), further corroborating the crystalline structure of the Tp‐BD(OH)_2_ COF membrane. In addition, surface zeta potential (Figure 2i) and water contact angle measurements (Figure 2j) imply that the COF membrane carries a large amount of strong negative charges and shows excellent hydrophilicity. Collectively, the highly ordered perpendicular channels with an opening of 1.91 nm in conjunction with the strong negative charges would endow the Tp‐BD(OH)_2_ COF membrane with superior size sieving and electrostatic hindrance selectivity in NF applications while the dual‐layer asymmetric structure and the great hydrophilicity could afford high water permeation rate.

### 2.2 Ultrafast and Precise Molecular Sieving in NF

**Figure 3:**
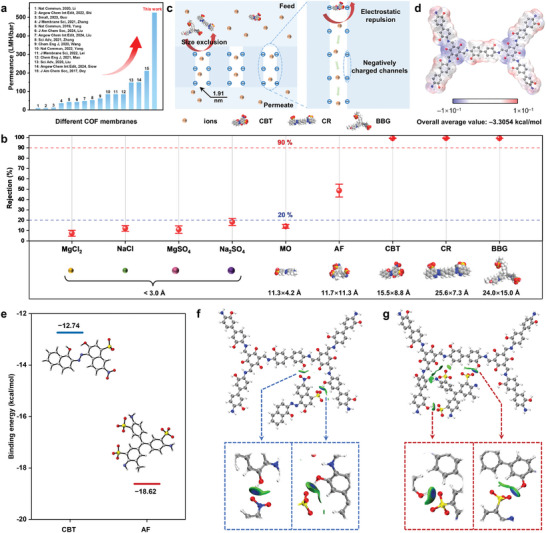
Nanofiltration performance and selective sieving mechanism of the Tp‐BD(OH)_2_ COF membrane. a) Comparison of pure water permeance with the recently reported COF membranes. b) Membrane rejections toward various inorganic salts and organic solutes with different molecular dimensions. The insets illustrate the molecular configurations and dimensional sizes of the solutes. c) Schematic illustration of the separation mechanism of the Tp‐BD(OH)_2_ COF membrane for precise separation of dyes and salt ions. d) Electrostatic potential (ESP) distribution and the obtained quantitative values of the COF molecular fragments based on DFT atomic calculations (scale bar unit: a.u.). e) The calculated binding energies of the COF molecular fragments with CBT and AF. The embedded images are the geometric configurations of CBT and AF optimized by DFT. f,g) Visualization of the interactions of f) CBT/COF configuration and g) AF/COF configuration obtained by the independent gradient model based on Hirshfeld partition (IGMH). The box below shows a magnified illustration of the local H‐bonding interactions.

The water filtration performance of the anisotropic Tp‐BD(OH)_2_ COF membrane was examined using a low‐pressure NF system under a wide spectrum of test conditions. Under the optimal synthesis conditions (Figures S8–S10, Supporting Information), the Tp‐BD(OH)_2_ COF membrane shows record‐breaking pure water permeance of 525.3 LMH bar^−1^, far exceeding the pure water permeance of most reported COF membranes (**Figure** **3**a; Table S4, Supporting Information). Meanwhile, the high water permeance was almost fully retained during the filtration of different solutes (Figure S11, Supporting Information). The obtained unprecedented water flux is imperative for improving the process efficiency and reducing the energy consumption of NF water separations. We posit that the ultra‐permeable properties of the Tp‐BD(OH)_2_ COF membrane mainly lean upon the synergistically combined merits of the advanced dual‐layer asymmetric structure and the highly ordered channels with a straight‐through AA stacking, which energetically facilitate the transport of water with low resistance.

The molecular sieving ability of the Tp‐BD(OH)_2_ COF membrane was then evaluated using various inorganic salts and dye molecules with distinct dimensions and charge features as the probe solutes. As shown in Figures 3b and S12 (Supporting Information), the COF membrane exhibits a sharp exclusion cut‐off between ions and dyes, where superior rejections of >99% are particularly achieved for eriochrome black T (CBT), congo red (CR), and brilliant blue G (BBG), while the rejection rates to both mono‐ and di‐valent ions (i.e., Na^+^, Mg^2+^, Cl^−^, and SO_4_^2−^) are below 20%. The facilitated transport of the cations and anions regardless of the charge and valence is ascribed to their small sizes, which are much smaller than the pore opening of the COF channels (i.e., 1.91 nm). In the same vein, the almost full rejection of CR and BBG is likely owing to their large molecular dimensions relative to the COF membrane pore size (Figure 3c; Table S5, Supporting Information). A correlation between dye rejection and the dimensional size of the corresponding dye molecule is identified in Figure S13 and Table S5 (Supporting Information), further underpinning the dominant role of the size exclusion mechanism.^[^ ^23^ ^]^ More specifically, methyl orange (MO) and acid fusion (AF) with a molecular dimension far smaller than the pore size of the COF membrane can easily diffuse through the membrane with low rejections. Electrostatic potential (ESP) distribution analysis (Figure S14, Supporting Information) reveals that the MO molecule shows an opposite ESP distribution at the two edges that bear a ─SO_3_^−^ and dimethyl group, respectively, which forces it to tend to permeate the COF membrane vertically, resulting in a relatively lower rejection in comparison to AF. Density functional theory (DFT) calculations also manifest the significant contribution of electrostatic repulsion to the efficient discrimination of CBT with a molecular dimension slightly smaller than that of the COF pore size.^[^ ^24^ ^]^ The ESP distribution of Tp‐BD(OH)_2_ COF molecular fragments (Figure 3d) indicates a negative overall average value of −3.3054 kcal mol^−1^, consistent with the measured zeta potential (Figure 2i). The ESP distributions (Figure S15, Supporting Information) of dye/COF‐fragment configurations in tandem with the calculated single‐point energies (SPE, Table S6, Supporting Information) confirm that the binding energies of COF molecular fragments with CBT and AF are −12.74 and −18.62 kcal mol^−1^, respectively (Figure 3e). The pronounced binding energy of the CBT/COF configuration implies that CBT is more likely to be repelled by the membrane. The independent gradient model of Hirshfeld's partition (IGMH) was further used to visualize the interactions of the COF configuration with CBT and AF.^[^ ^25^ ^]^ It turns out that the interaction region between AF and COF configuration is significantly larger than that of CBT (Figure 3f,g; Figure S16, Supporting Information). The two ─SO_3_^−^ groups on AF form H‐bonds with the COF configuration while the remaining ─SO_3_^−^ affords large‐area van der Waals interactions, which greatly reduces the system energy and thus achieves a stable energy configuration. On the contrary, such an energetic stable configuration is hard to achieve for CBT as only H‐bonds exist between the COF configuration and ─NO_2_ and ─SO_3_^−^ on CBT. Molecular polarity index (MPI) was further calculated to understand the impact of H‐bonding and electrostatic interactions. The obtained MPI of Tp‐BD(OH)_2_ COF membrane, CBT, and AF are 18.4, 58.3, and 172.6 kcal mol^−1^, respectively (Figure S17, Supporting Information). Compared with AF, CBT has a lower MPI, indicating that the COF membrane has a weaker affinity for it and therefore exhibits a higher rejection. Additionally, adsorption results (Table S7, Supporting Information) demonstrate that the dye adsorption rates are all below 10%, confirming that the excelled dye/ion sieving ability of the COF membrane mainly stems from the precisely regulated size exclusion and electrostatic repulsion effects.

**Figure 4:**
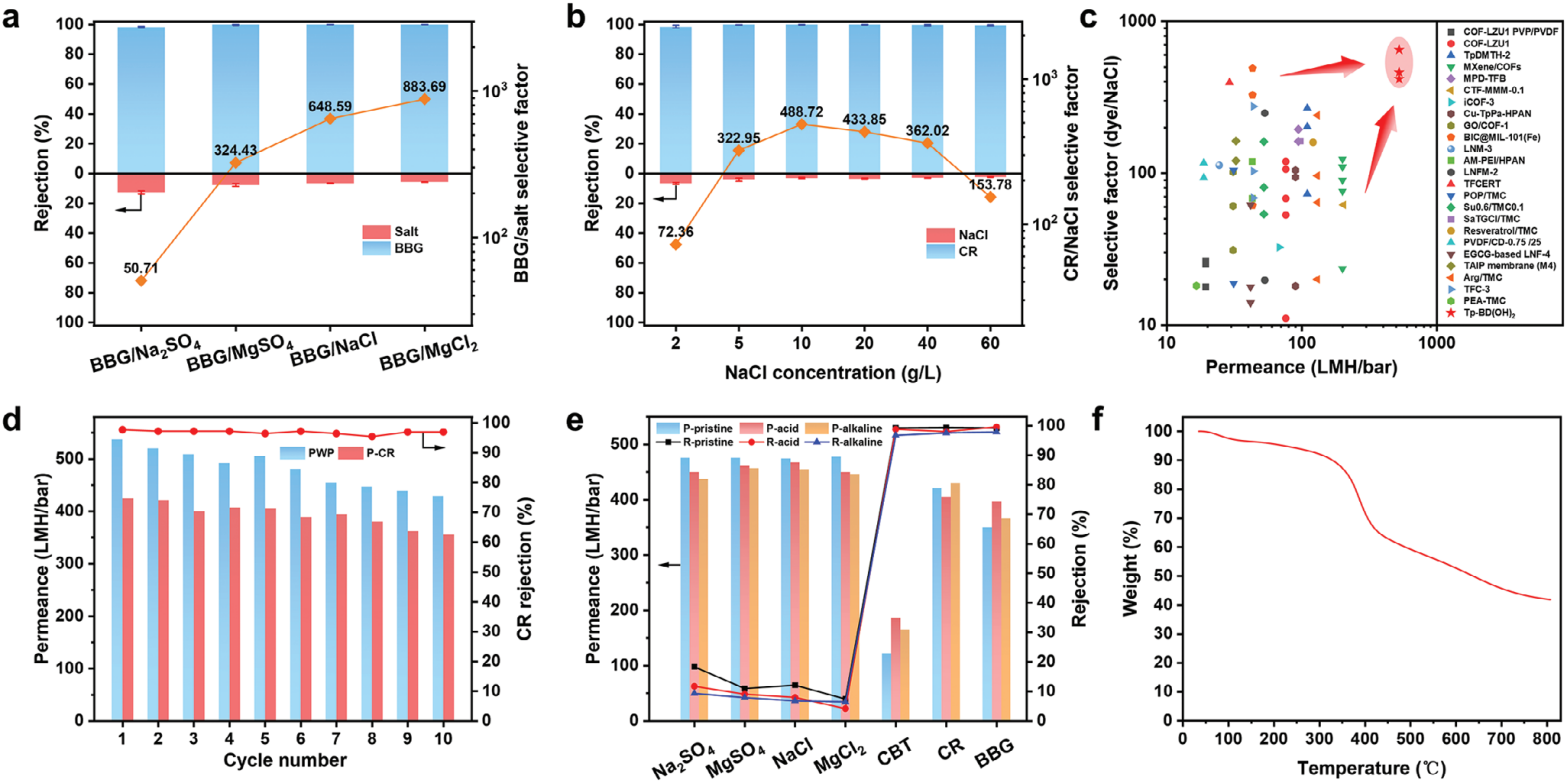
Nanofiltration performance for binary mixture systems and structural stability of the Tp‐BD(OH)_2_ COF membrane. a,b) Membrane molecular sieving performance for a) BBG/salts and b) CR/NaCl binary mixtures with different salts and NaCl contents. (Salt concentration: 2.0 g L^−1^ in a) and 2.0–60.0 g L^−1^ in b), dye concentration: 50.0 mg L^−1^ in a,b), operation pressure: 1.5 bar). The red and blue columns refer to the salt and dye rejection respectively, and the orange polyline represents the corresponding dye/salt selective factor. c) Molecular sieving performance comparison of the Tp‐BD(OH)_2_ COF membrane with state‐of‐the‐art NF membranes. The corresponding references are specified in Table S8 (Supporting Information). d) Structural durability of the Tp‐BD(OH)_2_ COF membrane. The blue and red columns refer to the water permeance for filtrating pure water and CR solution, respectively, while the red polyline represents the CR rejection. e) Chemical stability of the Tp‐BD(OH)_2_ COF membrane. The blue, red, and orange columns represent the water permeance of the pristine membrane and the membranes after being exposed to strong acid and alkali, respectively. The black, red, and blue polylines stand for the corresponding rejections toward different solutes. f) Thermal stability of Tp‐BD(OH)_2_ COF membrane. Thermogravimetric analysis (TGA) curve of the membrane in nitrogen atmosphere.

We further studied the separation performance of the Tp‐BD(OH)_2_ COF membrane for binary mixtures containing different dyes and salts. Taking the BBG/salts binary mixtures as representative examples (**Figure** **4**a; Figure S18, Supporting Information), the selective factors of each mixture made by BBG and Na_2_SO_4_, MgSO_4_, NaCl, and MgCl_2_ reach 50.7, 324.4, 648.6, and 883.7, respectively, along with impressively high water permeance of over 380 LMH bar^−1^, far exceeding the levels of other NF membranes reported so far. Similar performances were also achieved by the COF membrane for the binary mixtures of CBT/CR and various salts (Figures S19 and S20, Supporting Information). Feed streams in actual water treatment often contain a wide range of salt and dye contents, which may alter membrane performance, especially at high concentrations, owing to the electrostatic screening effects in NF. Remarkably, Figures 4b and S21–S22 (Supporting Information) show that the COF membrane maintains its superior dye/salt (i.e., CR/NaCl) selectivity and water flux across a wide range of feed salt content from 2.0 to 60.0 g L^−1^ and dye concentration from 10.0 to 200.0 mg L^−1^, signifying its strong electrostatic shielding resistance toward high ionic strength, which likely results from the superior size sieving ability of the COF membrane exerted by its highly ordered channels. Compared with previously reported COF/metal–organic framework (MOF) films and state‐of‐the‐art polymeric membranes (Figure 4c; Table S8, Supporting Information), the Tp‐BD(OH)_2_ COF membrane exhibits superior advances in both water permeance (larger than 500 LMH bar^−1^) and dye/salt selectivity (over 410), underscoring the great merits of the synthetically engineered anisotropic COF membrane structure. In addition, the Tp‐BD(OH)_2_ COF membrane exhibits excellent structural durability, where the average water permeance was maintained at 481.3 LMH bar^−1^ with a constant CR rejection of 96.9% throughout the long‐term cycle tests (Figure 4d). Figures 4e and S23 (Supporting Information) further show that the membrane permselectivity and chemical structure were retained after exposing the membrane to strong acid and alkaline environments, demonstrating its robust chemical stability, which is far superior to MOF and traditional polymeric membranes. Furthermore, the COF membrane has good thermal stability (Figure 4f). These compelling results unequivocally demonstrate the great potential of the Tp‐BD(OH)_2_ COF membrane for precise and efficient molecular sieving under various circumstances close to practical applications.

### 2.3 Mechanistic Insights into the Interface‐Confined Catalytic Synthesis

The synthetic construction of the anisotropic Tp‐BD(OH)_2_ COF membrane exemplifies the great potential of interface‐confined catalysis in the precision regulation of COF membrane microstructure and its separation performance. To extend the application of this distinctive strategy, it is crucial to understand the underlying mechanism responsible for the formation of the unusual dual‐layer cross‐section structure in the Tp‐BD(OH)_2_ COF membrane. Inspired by the prestigious confined catalysis, our approach is contingent on the bidirectional diffusion of COF precursors and the catalyst at the interface as well as leverages the strong attractive interactions between the COF fragments and catalyst to enable an interface‐confined catalytic polycondensation. To realize this exquisite design strategy, Tp‐BD(OH)_2_ COF with abundant ─OH groups and β‐ketoenamine bonds were purposely designed as they can substantially capture and anchor the HAc catalyst through the H‐bonding interactions at the interface of cyclohexanone and water (Figure 1a).

**Figure 5:**
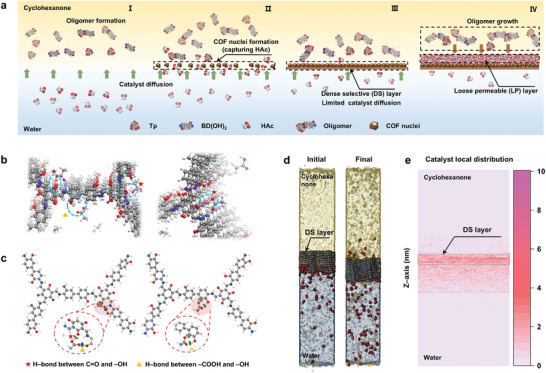
Mechanistic analysis of the interface‐confined catalytic synthesis strategy. a) Schematic illustration of the formation process of anisotropic Tp‐BD(OH)_2_ COF membrane with a dual‐layer asymmetric cross‐sectional microstructure. b) Schematic visualization of the H‐bonding interactions between COF fragments and HAc catalyst. The section enclosed by the blue dashed circular frame indicates the formed H‐bonds. c) Schematic visualization of the interactions in the HAc/COF‐fragment configuration obtained by independent gradient model based on Hirshfeld partition (IGMH). The red dashed circular frame shows a magnified image of the local H‐bonding interaction. d) Molecular dynamics (MD) simulation snapshots of HAc diffusion from the aqueous phase across the interface into the cyclohexanone phase at 0 and 17 000 ps in a system with a COF dense selective (DS) layer at the interface. e) The obtained average density distribution of HAc along the Z‐axis (frontal view) simulated within 20 ns for the system with a dense COF layer at the interface (scale bar unit: counts).

As illustrated in **Figure** **5**a, the formation of a Tp‐BD(OH)_2_ COF membrane with an anisotropic microstructure includes four stages. In the initial stage of IP (stage I), Tp and BD(OH)_2_ precursors dissolved in the upper cyclohexanone oil phase polymerize to form COF oligomers, while the HAc catalyst in the bottom aqueous phase diffuses toward the water/cyclohexanone interface. When the oligomers meet with HAc at the interface, COF nuclei are rapidly formed through the catalyzed polycondensation reaction and gradually agglomerate (stage II). With the formation of COF aggregates at the interface, the ─OH and β‐ketoenamine moieties on the Tp‐BD(OH)_2_ frameworks form H‐bonds with HAc, capturing and confining a large amount of HAc to an interfacial reaction zone, which facilitates COF crystallization and the formation of a relatively dense and crystalline nascent COF nanofilm at the incipient stage of polycondensation (stage III). The formed dense COF film in tandem with its strong chemical interactions with HAc, in turn, hinders the subsequent diffusion of HAc toward the oil phase, in which the depleted HAc induces an inefficient polycondensation of the precursors and thus the formation of a loose permeable layer (stage IV), leading to the successful construction of a self‐sustaining COF membrane with an asymmetric dual‐layer structure.

To gain fundamental insights into the proposed membrane formation mechanism at the molecular level, DFT calculations along with the corresponding IGMH analysis were first employed to reveal the profound H‐bonding interactions between the Tp‐BD(OH)_2_ COF and HAc catalyst. As illustrated in Figures 5b,c and S16 (Supporting Information), two types of O─H···O═C H‐bonds with a bond length of ≈1.59 and 1.77–1.83 Å are unequivocally formed either between C═O of the β‐ketoenamine and the ─OH on HAc or between the ─OH on BD(OH)_2_ and the C═O of HAc, which are in good agreement with our conjecture. Owing to the strong directionality of these H‐bonding interactions, HAc is firmly anchored onto the β‐ketoenamine moieties and ─OH groups on the COF nuclei during stages II and III, effectively catalyzing the polycondensation reaction and thus facilitating the formation of a dense and crystalline COF thin layer at the initial stage of IP. In turn, the formed dense COF layer significantly hinders the diffusion of HAc at the interface, which results in a substantial decrease of the HAc concentration in the oil phase that contains the COF‐forming precursors, making effective polycondensation reaction difficult to occur, thereby forming a highly permeable sublayer. Molecular dynamics (MD) simulations firmly corroborate the retarded diffusion of HAc molecules at the interface in the presence of a nascent COF layer compared to a free interface (Figure 5d; Figure S24a, Supporting Information).^[^ ^26^ ^]^ The calculated average density distribution of HAc along the z‐axis direction (frontal view) indicates that the average density of HAc at the interface with a relatively dense COF layer is much higher than that in the oil phase as most of the HAc molecules are captured and anchored in the Tp‐BD(OH)_2_ frameworks, leading to a substantial enrichment of HAc at the interface while inhibiting its subsequent diffusion into the oil phase to collide with the precursors (Figure 5e; Figure S24b, Supporting Information). Meanwhile, MD results demonstrate that a considerable number of hydrogen bonds are formed between the COF structure and HAc (Figure S25, Supporting Information), implying that the enrichment of HAc at the interface is largely dependent on the formation of H‐bonding interactions. In contrast, more HAc diffuses through the interface into the oil phase in the system without a dense COF layer, and the average HAc density at the water/cyclohexanone interface is substantially lower than the oil phase. DFT and MD molecular simulations validate the underlying mechanisms of the innovative interface‐confined catalytic synthesis strategy implemented in this study, which holds broad prospects for the rational design and synthesis of anisotropic COF membranes with unparalleled separation properties.

## 3 Conclusion

Facile and robust interfacial synthesis of polycrystalline COF membranes with both high water permeation flux and precision molecular sieving capabilities remains a big challenge. Taking inspiration from the prestigious idea of confined catalysis, this work develops an innovative interface‐confined catalytic synthesis strategy toward molecular construction of anisotropic COF membrane with a dual‐layer asymmetric cross‐section microstructure to overcome the permeance/selectivity trade‐off in energy‐efficient NF for molecular fractionation. MD simulations and DFT calculations reveal that our approach is contingent on exquisite control over the bidirectional diffusion of the precursors and acid catalyst as well as the judicious manipulation of the chemical interactions between COF segments and the catalyst. More specifically, the β‐ketoenamine bonds and ─OH groups in the exemplified Tp‐BD(OH)_2_ COF substantially capture and enrich the HAc catalyst through H‐bonding interactions, which instigates confined catalytic polycondensation between the aldehyde and amine precursors, facilitating the rapid formation of COF nuclei and the subsequent growth of a nascent COF crystalline layer at the incipient stage of membrane formation. The formed dense layer, in turn, restricts the subsequent diffusion of acid catalyst across the interface toward the precursors and thus retard the subsequent formation of crystalline COF structures, leading to in situ formation of an anisotropic Tp‐BD(OH)_2_ COF membrane during one‐step interfacial polymerization.

The dual‐layer structure affords the Tp‐BD(OH)_2_ membrane unparalleled separation performance in textile water desalination, with record‐breaking pure water permeance of 525.3 LMH bar^−1^, significantly higher than those of previously reported COF membranes, and superior dye/salt selective factor of up to 648.6, which is far superior to state‐of‐the‐art NF membranes reported to date. Experimental results and DFT calculations confirm that the polycrystalline COF dense layer possesses precisely regulated pore sizes and charge characteristics that allow the ultrafast permeation of water molecules and salt ions but energetically block the organic molecules through a synergetic mechanism of steric hindrance and electrostatic repulsion. In addition, the Tp‐BD(OH)_2_ COF membrane exhibits outstanding structure durability and chemical stability in both strong acid and alkaline environments. The reported interface‐confined catalysis strategy sheds light on the rational design and synthesis of advanced COF nanofilms with anisotropic microstructure via one‐step interfacial polycondensation, accelerating the development of high‐performance NF membranes with the competency of ensuring precision molecular sieving in widespread applications related to water and energy.

## 4 Experimental Section

### Synthesis of Anisotropic Tp‐BD(OH)_2_ COF Membranes

The self‐sustaining Tp‐BD(OH)_2_ COF membranes were synthesized via an optimized interface‐confined catalytic interfacial polymerization approach at room temperature under autogenous pressure. In a typical synthesis, 1,3,5‐triformylphoroglucinol (Tp, 0.15 mmol) and 3,3′‐dihydroxybenzidine (BD(OH)_2_, 0.225 mmol) were dissolved in cyclohexanone (50 mL) as the oil phase, and 3.0 mol L^−1^ acetic acid (HAc, 1.2 mL) in ultrapure water (50 mL) served as the aqueous phase. The cyclohexanone solution was added to the HAc solution to form a stable interface, allowing a continuous COF membrane to form after five days. The membrane was then cleaned with cyclohexanone, ethanol, and water to remove residual monomers and HAc. The synthesized COF membrane was transferred to a polysulfone (PSf) substrate for nanofiltration performance testing. The detailed procedures for the synthesis of Tp‐BD(OH)_2_ COF membranes and characterization are disclosed in the Supporting Information.

### Molecular Dynamics (MD) Simulations

MD simulations were performed using GROMACS 2020.6 software.^[^ ^26^ ^]^ Energy minimization was conducted with the steepest descent algorithm to reduce steric clashes, aiming for a force convergence of <100 kJ mol^−1^ nm^−1^. Simulations used the leap‐frog algorithm with a 1 fs time step and a 20 ns duration. The system temperature was maintained at 298.15 K using the V‐rescale thermostat, while isotropic pressure coupling was applied via the Berendsen barostat. Long‐range electrostatics were treated with the PME method, and van der Waals interactions were truncated at 1.2 nm. The LINCS algorithm ensured bond constraints. Periodic boundary conditions were applied. RESP charges for oligomers, solvent, and acetic acid molecules were calculated using ORCA (Version 5.0.4) with the B3LYP functional and def2‐TZVP basis set,^[^ ^24^, ^27^ ^]^ and analyzed using Multiwfn.^[^ ^24^, ^28^ ^]^ The detailed specifications of MD simulations are provided in the Supporting Information.

### Density Functional Theory (DFT) Calculations

Quantum chemical calculations were performed to analyze the chemical properties of COF fragments, dye molecules, and their interactions with COF channels using ORCA software (Version 5.0.4).^[^ ^24d^ ^]^ Conformational searches were done with the gfn2‐xtb method in xtb software, yielding 100 conformations, which were optimized and energy‐calculated. Structures within 1 kcal mol^−1^ and 1 Å were considered identical. DFT calculations used the B3LYP functional with D3BJ dispersion corrections,^[^ ^27^ ^]^ and geometry optimizations were performed with the B97‐3c functional.^[^ ^24^, ^29^ ^]^ The SMD solvation model was applied.^[^ ^30^ ^]^ Binding energies were calculated as *E*_binding_ = *E*_total_ – *E*_0_ – *E*_1_. ESP analysis and interaction studies were done using Multiwfn and IGMH,^[^ ^24^, ^25^, ^28^ ^]^ with visualizations created in VMD (Version 1.9.3). Specific DFT calculation details can be found in the Supporting Information.

### Membrane Separation Performance Tests

The separation performance of the Tp‐BD(OH)_2_ COF membrane was evaluated under various test conditions using a high‐pressure stirred permeation cell in nanofiltration mode. The pure water permeance of the membrane was measured using deionized water, and the solute rejection tests were performed using inorganic salts (i.e., NaCl, MgCl_2_, MgSO_4_, and Na_2_SO_4_) and dye molecules (i.e., Methyl orange, MO; Acid fusion, AF; Eriochrome black T, CBT; Congo red, CR; and Brilliant blue G, BBG) as the solutes. The dye/salt selective factor was determined using binary mixtures consisting of different amounts of dye and inorganic salt as the feed. Long‐term stability tests were conducted using 50.0 mg L^−1^ CR solution as the feed solution for 10 cycles. Chemical stability was assessed by soaking the COF membrane in 38 wt.% HCl or 0.4 wt.% NaOH solution for 10 days, respectively. The adsorption rate of different dyes on the COF membrane was measured by soaking the membrane in 50.0 mg L^−1^ dye solution. The detailed experimental procedures, operating conditions, and the determination of water permeance, rejection, and selective factor are described in the Supporting Information.

## Conflict of Interest

The authors declare no conflict of interest.

## Supporting information



Supporting Information

## Data Availability

The data that support the findings of this study are available from the corresponding author upon reasonable request.
