# Supplementary material for: Interface‐Confined Catalytic Synthesis of Anisotropic Covalent Organic Framework Nanofilm for Ultrafast Molecular Sieving
Source: Adv Sci (Weinh). 2025 Feb 20;12(15):2415520. doi: 10.1002/advs.202415520 (PMC12005809; doi:10.1002/advs.202415520)
Supplement: Supplementary file 1 — Supporting Information [file ADVS-12-2415520-s001.pdf]

ADVANCED SCIENCE
Open Access

Supporting Information

for *Adv. Sci.*, DOI 10.1002/advs.202415520

Interface-Confined Catalytic Synthesis of Anisotropic Covalent Organic Framework Nanofilm for Ultrafast Molecular Sieving

*Jiahao Tang, Yu Liao, Zhenxiang Pan, Songjun Fang, Mingxiu Tang, Lu Shao and Gang Han**

Supporting Information

Interface-confined Catalytic Synthesis of Anisotropic Covalent Organic Framework Nanofilm for Ultrafast Molecular Sieving

Jiahao Tang [a], Yu Liao [a], Zhenxiang Pan [a], Songjun Fang [a], Mingxiu Tang [a], Lu Shao [b], Gang Han [a, *]

[a] College of Environmental Science and Engineering, Tianjin Key Laboratory of Environmental Remediation and Pollution Control, Nankai University, 38 Tongyan Road, Tianjin, 300350, China

[b] MIIT Key Laboratory of Critical Materials Technology for New Energy Conversion and Storage, State Key Laboratory of Urban Water Resource and Environment (SKLUWRE), School of Chemistry and Chemical Engineering, Harbin Institute of Technology, Harbin 150001, China

* Corresponding author

Tel: +86 022-23501117. Email: hangang@nankai.edu.cn

- Number of Pages: 54
- Number of Figures: 25
- Number of Tables: 8

1. Experimental and Methods

1.1 Chemicals and materials

1,3,5-Triformylphoroglucinol (Tp, 97%) and 3,3'-Dihydroxybenzidine ($BD(OH)_2$, 98%) were purchased from Zhengzhou Alpha Chemical Co., Ltd. Sodium chloride (NaCl, 99.8%), sodium sulfate anhydrous (Na_2SO_4, 99%), magnesium chloride hexahydrate ($MgCl_2 \cdot 6H_2O$, 99%), magnesium sulfate anhydrous ($MgSO_4$, AR), methyl orange (MO, 98%), acid fuchsin (AF, 70%), eriochrome black T (CBT, biotechnology grade), congo red (CR, biodye), brilliant blue G (BBG, AR), cyclohexanone (Anone, 99%), and acetic acid (HAc, 99.5%) were obtained from Shanghai Macklin Biochemical Co., Ltd. Sodium hydroxide (NaOH, 99.9%) was provided by Shanghai Aladdin Biochemical Technology Co., Ltd. Hydrochloric acid (HCl, AR) was ordered from Fuchen (Tianjin) Chemical Reagent Co., Ltd. Commercial polysulfone (PSf) membrane substrate was purchased from Hunan Keensen Technology Co., Ltd. Ultra-pure water (18.2 MΩ·cm) was generated by a Millipore-D 24 UV ultrapure water integrated system (Millipore Instrument, USA). Unless otherwise specified, all chemicals and reagents were used as received without further purification.

1.2 Synthesis of Tp-$BD(OH)_2$ COF membranes

The self-sustaining Tp-$BD(OH)_2$ COF membranes were synthesized via a synergistically optimized interfacial polymerization approach at room temperature under autogenous pressure. In a typical synthesis, Tp (0.15 mmol, 31.5 mg) and $BD(OH)_2$ (0.225 mmol, 48.7 mg) were dissolved in 50 mL cyclohexanone and used as the oil phase. 1.2 mL 3.0 mol L^{-1} HAc was mixed with 50 mL ultrapure water as the aqueous phase. Subsequently, the cyclohexanone

solution containing the precursors was slowly added to the top of the HAc aqueous solution to form a stable cyclohexanone/water interface in a reactor, which was then sealed with a parafilm and kept undisturbed. A continuous and stable self-sustaining Tp-BD$(OH)_2$ COF membrane was formed at the interface after five days of reaction. When the reaction was complete, the top oil phase and bottom aqueous phase were carefully drained and the synthesized COF membrane was transferred to a petri dish for cleaning. The membrane was then thoroughly rinsed with cyclohexanone, ethanol, and ultra-pure water three times each to remove the residual monomers and acid catalyst. Using the same synthetic procedure, condition optimization experiments were performed by varying reaction time and reactants/HAc concentrations to determine the optimum conditions for high-quality membranes. Finally, the as-synthesized Tp-BD$(OH)_2$ COF membrane was transferred to the PSf substrate for nanofiltration performance tests.

1.3 Characterization

Fourier-transform infrared (FT-IR) spectra were acquired on a Nicolet iS50 Fourier transform infrared spectrometer (Thermo Scientific, USA) in attenuated total reflection (ATR) mode. Each sample was measured for a total of 32 scans with a resolution of 4 cm^{-1} in the spectral range of 800–4000 cm^{-1}. Elemental and chemical spectroscopic analyses were determined by X-ray photoelectron spectroscopy (XPS) using a Thermo Fisher ESCALAB Xi+ instrument with a monochromic Al-Kα X-ray source (hν = 1486.6 eV). High-resolution scans of the C, N, and O regions were performed at 0.5 eV increments, and the obtained XPS peaks were fitted using Advantage software. The element distribution of the membrane was examined by energy-

dispersive X-ray spectroscopy (EDS, TESCAN MIRA LMS, Czech Republic). The membrane surface and cross-section morphology were characterized by field emission scanning electron microscopy (FESEM, JSM-7800F, JEOL Asia Pte Ltd, Japan) and transmission electron microscopy (TEM, FEI-Talos F200S, Thermo Scientific, USA). FESEM samples were sputter-coated with gold/palladium alloy using a Desk II cold sputter unit (Denton Vacuum LLC) to prevent charging. In EDS and FESEM characterization, the accelerating voltage was 3 kV for morphology characterization, 15 kV for spectrum mapping shooting, and the detector was an SE2 secondary electron detector. An atomic force microscope (AFM, Bruker Dimension ICON, Germany) was employed to examine the membrane surface topography under the tapping mode. For each sample, a scanning area of 10 × 10 μm was measured and the roughness average (Ra) was then calculated and reported. The membrane crystalline structure was evaluated by high-resolution transmission electron microscopy (HRTEM, FEI-Talos F200S, Thermo Scientific, USA) and X-ray diffraction (XRD, Ultima IV, Rigaku Corporation, Japan) measurements over an angular range of 0.5–40° (2θ) with a step size of 1° per minute at ambient conditions. Surface water contact angles were measured at ambient temperature with the sessile drop method on a Contact Angle Goniometer (WCA, Kruss DSA23S, Germany) using deionized water. At least ten readings were taken at random spots on each sample and the average value was reported. Surface zeta potential measurements were performed on an electrokinetic analyzer (SurPASS 3, Anton Paar, Austria) with a background electrolyte of 1 mM KCl solution, and the pH was adjusted using HCl and NaOH buffer solutions. Thermogravimetric analysis (TGA) was conducted on a TA Instruments 550 thermogravimetric analyzer in a nitrogen atmosphere over a temperature range of 30–800 °C with a heating rate of 20 °C min^{-1}. The N_2 adsorption–

desorption isotherms and pore size distribution were measured on a Micromeritics ASAP 2460 apparatus at 77 K. The Brunauer–Emmett–Teller (BET) surface area was calculated over a range of relative pressures between 0.05 and 0.20, and the pore size distribution was analyzed based on a QSDFT model.

1.4 Nanofiltration molecular sieving performance tests

The nanofiltration (NF) performances of the Tp-BD$(OH)_2$ COF membrane was evaluated at room temperature using a stirred stainless-steel dead-end cell with an effective filtration area of 7.9×10^{-5} m^2. Before collecting data, the membrane sample was conditioned at a pre-determined pressure for at least 40 min to attain a steady flux. The water permeance (P, L m^{-2} h^{-1} bar^{-1}) was then measured at 1.5 bar using deionized water as the feed, and the value of P was calculated by Eq. (S1).

$$P = \frac{V}{A \times \Delta t \times \Delta p} \qquad (S1)$$

where V (L) is the volume of permeate across the membrane collected during a time interval of Δt (h), A (m^2) is the effective filtration area, and Δp (bar) is the trans-membrane hydraulic pressure.

Membrane selectivity was examined via rejection tests that were performed under various testing conditions by using different types of solutes. More specifically, NaCl, $MgCl_2$, $MgSO_4$, Na_2SO_4, MO, AF, CBT, CR, and BBG solutions with different concentrations and compositions were used as the feed. For single-solute NF tests, 50.0 mg L^{-1} dye solution and 2.0 g L^{-1} salt solution were used as the feed, respectively, while in the binary-mixture tests, the dye

concentration varied from 10.0 to 200.0 mg L^{-1} and the salt concentration ranged from 2.0 to 60.0 g L^{-1}. To minimize the interferences of external concentration polarization, a high stirring speed of 600 rpm was used during the tests. For each test, the membrane rejection R (%) was calculated by Eq. (S2).

$$R = \left(1 - \frac{C_p}{C_f}\right) \times 100\% \qquad (S2)$$

where C_p and C_f are the solute concentrations of the permeate and feed solution, respectively. The dye content in the solution was measured by a UV-vis spectrophotometer (UV-1601, Beijing Beifen-Ruili Analytical Instrument (Group) Co., Ltd, China). The salt concentration in the permeate and feed was obtained by conductivity measurement using a SevenCompact™ S230 (Mettler Toledo) conductivity meter. The selective factor of a binary-mixture solution was calculated by Eq. (S3).

$$SF = \frac{1 - R_{salt}}{1 - R_{dye}} \qquad (S3)$$

where R_{salt} and R_{dye} are the rejections of salt and dye, respectively. For each measurement, at least three membrane samples were tested to reduce experimental error and ensure data reproducibility.

To test the long-term cycle stability of the membrane, the membrane sample was first conditioned at a pre-determined pressure for at least 40 min to reach a stable flux. Then, the water permeance and CR rejection of each cycle were measured following the protocol described above. Between each test, the residual CR adsorbed on the membrane and filtration cell was thoroughly cleaned with methanol and water. For membrane chemical stability tests, the membrane sample was soaked in 38 wt% HCl and 0.4 wt% NaOH solution with a pH value

of 12 for 10 days, respectively. After rinsing with ethanol and water three times each to fully remove the residual acids and bases, the separation performance of the membrane was tested and compared with a pristine one. The dye adsorption measurements were conducted by immersing the membrane sample in a dye solution for 72 hours without disturbance. Then, the absorbance of the solution before and after adsorption was measured to obtain the dye adsorption rate.

1.5 Molecular dynamics (MD) simulations

MD simulations were performed using GROMACS 2020.6 software. [1] Before simulations, energy minimization was first conducted to reduce any steric clashes and high-energy interactions in the initial structures using the steepest descent algorithm with the convergence criterion set to a maximum force of less than 100 kJ mol^{-1} nm^{-1}. After the energy minimization, MD simulations were carried out using the leap-frog algorithm with a time step of 1 fs and a total simulation time of 20 ns. The system temperature was controlled using the V-rescale thermostat with the reference temperature set to 298.15 K and a coupling time constant of 0.1 ps for the entire system. The system was subjected to isotropic pressure coupling using the Berendsen barostat, with a pressure coupling time constant of 2.0 ps and a compressibility of 4.5×10^{-5} bar^{-1}. Long-range electrostatics were treated using the Particle Mesh Ewald (PME) method with fourth-order interpolation and a Fourier grid spacing of 0.16 nm. Both short-range electrostatic and van der Waals interactions were truncated at a cut-off distance of 1.2 nm. The LINCS algorithm was employed to maintain bond constraints, and no additional constraints were applied to bond vibrations. Periodic boundary conditions (PBC) were applied in all three

spatial dimensions to simulate an infinite system. To account for the influence of atomic charges in molecular dynamics simulations, the RESP charges for oligomers and solvent molecules, as well as acetic acid molecules, were calculated by ORCA (Version 5.0.4). [2] The B3LYP functional with D3BJ dispersion corrections and the def2-TZVP basis set were employed for the optimization and single-point energy calculations of the aforementioned molecules. [3] The obtained results were analyzed using Multiwfn to extract the RESP charges. [4]

1.6 Density functional theory (DFT) calculations

The chemical properties of COF fragments and dye molecules as well as the mutual interactions of solutes with the COF channels were analyzed using quantum chemical calculations. All DFT atomic computations were performed using ORCA quantum chemistry software (Version 5.0.4). [2] Conformational searches were performed using the gfn2-xtb method in the xtb software, yielding 100 conformations that were subsequently optimized and their energies were calculated. Structures with similar energies (i.e., within 1 kcal mol^{-1}) and similar geometries (i.e., within 1 Å) were classified as identical. The B3LYP functional with D3BJ dispersion corrections was used for all calculations, [3] where geometry optimizations and single-point energy calculations were performed using the B97-3c functional and the def2-TZVP basis set, respectively. [5] The SMD solvation model was applied throughout the calculations. [6] Binding energies were determined by the equation $E_{\text{binding}} = E_{\text{total}} - E_0 - E_1$, where E_{total} is the total energy of the COF fragment with the dye molecule, E_0 is the energy of the COF fragment, and E_1 is the energy of the dye molecule. Electrostatic potential (ESP) analysis and

intermolecular/intramolecular interaction studies were performed with the Multiwfn software using the independent gradient model based on Hirshfeld partition (IGMH). [4] IGMH is an enhanced version of the IGM model that takes into account the electronic structure and chemical environment for more accurate analysis. [7] IGMH isosurface images were generated at an isovalue of 0.004. All visualizations were created using Visual Molecular Dynamics (VMD, Version 1.9.3).

2. Supplementary Figures

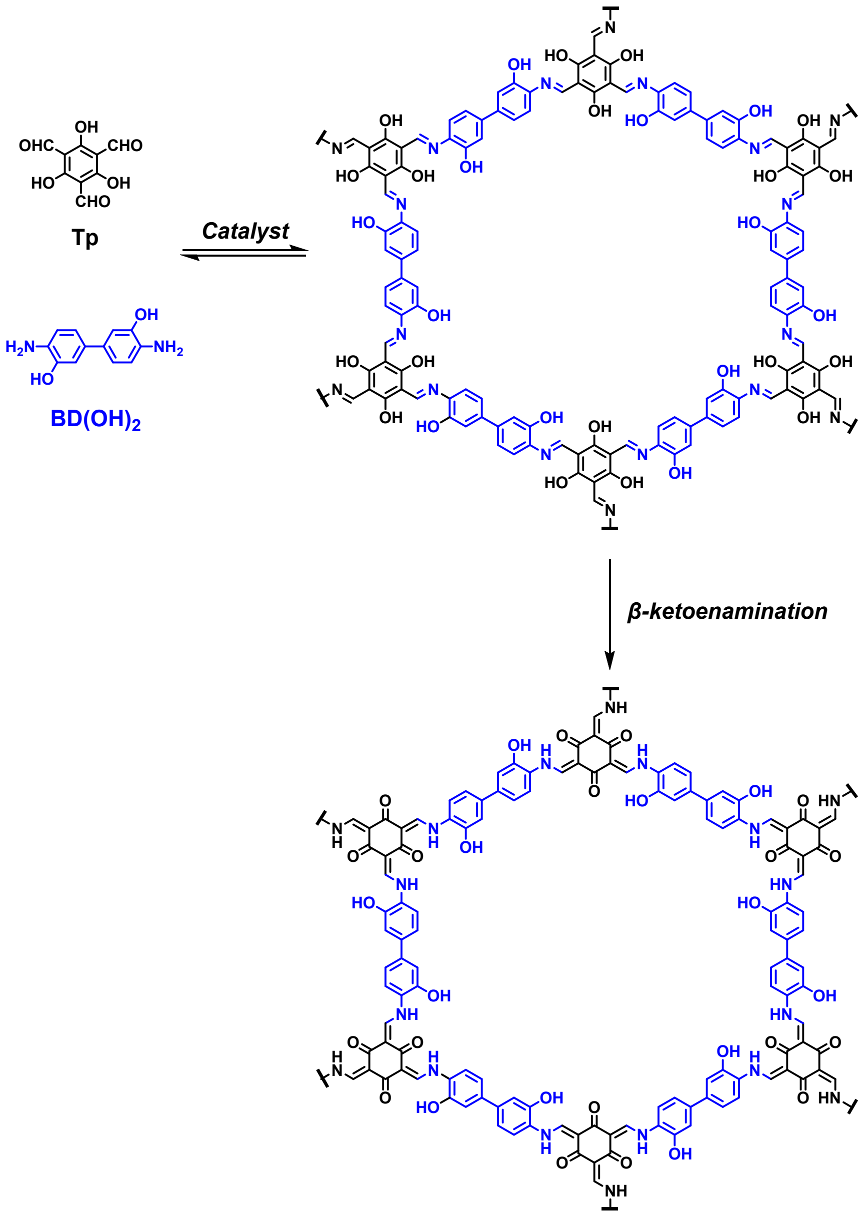

Figure S1. Schematic representation of the chemical synthesis route of Tp-BD(OH)$_2$ COF membrane. The synthesis mainly involves two steps: 1) Tp and BD(OH)2 precursors undergo dehydration polycondensation through imine bonds under the catalysis of Hac; and 2) β-ketoenamination forms the final Tp-BD(OH)$_2$ COF structure.

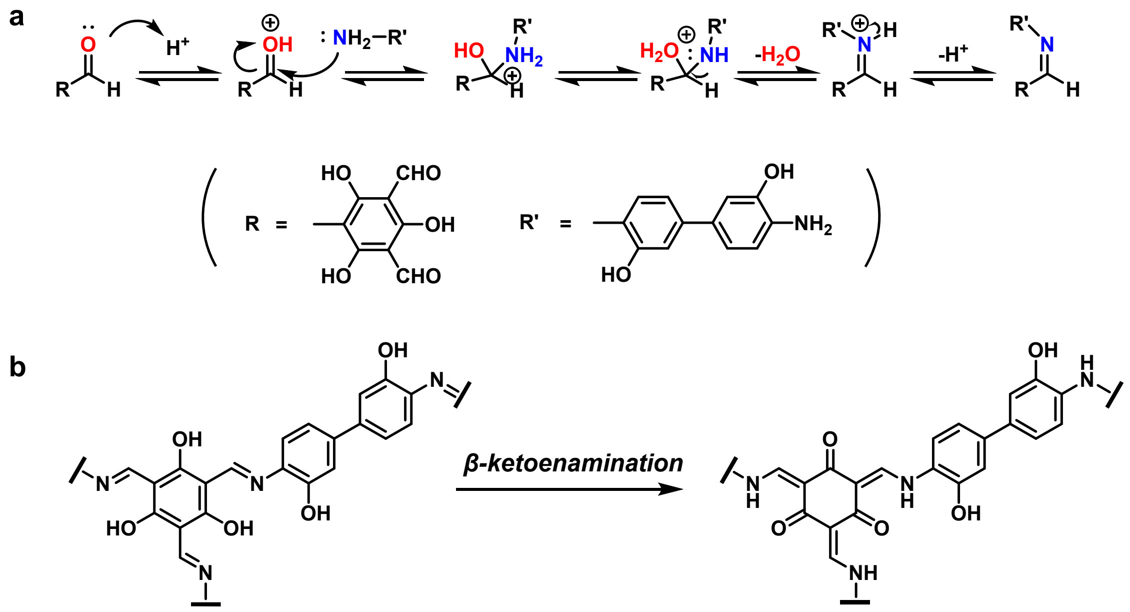

Figure S2. Chemical reaction mechanism for the synthesis of Tp-BD$(OH)_2$ COF membrane. (**a**) Reaction mechanism for the formation of imine bonds between Tp aldehyde and BD$(OH)_2$ amine in the presence of an acid catalyst. The lone electron pair on the amine nitrogen first attacks the proton-activated carbonyl carbon, triggering a nucleophilic addition and the formation of a hemiaminal intermediate, followed by the elimination of a water molecule. (**b**) Reaction mechanism for the β-ketoenamination of Tp-BD$(OH)_2$ COF structure.

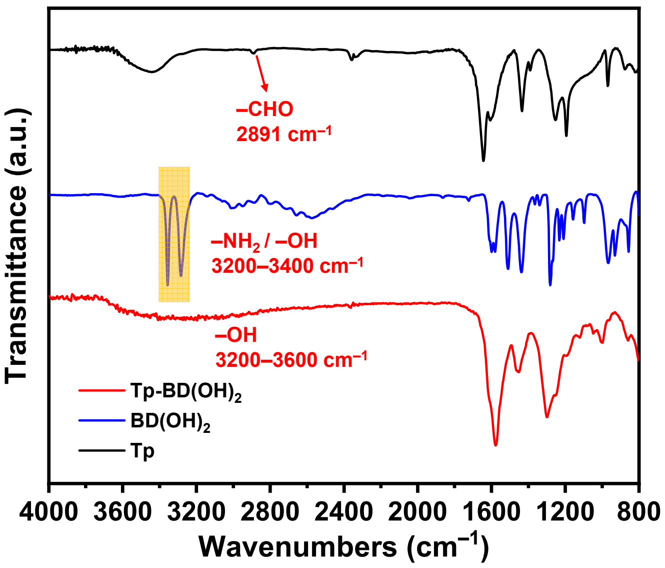

Figure S3. FT-IR spectra of Tp aldehyde and $BD(OH)_2$ amine precursors and Tp-$BD(OH)_2$ COF membrane.

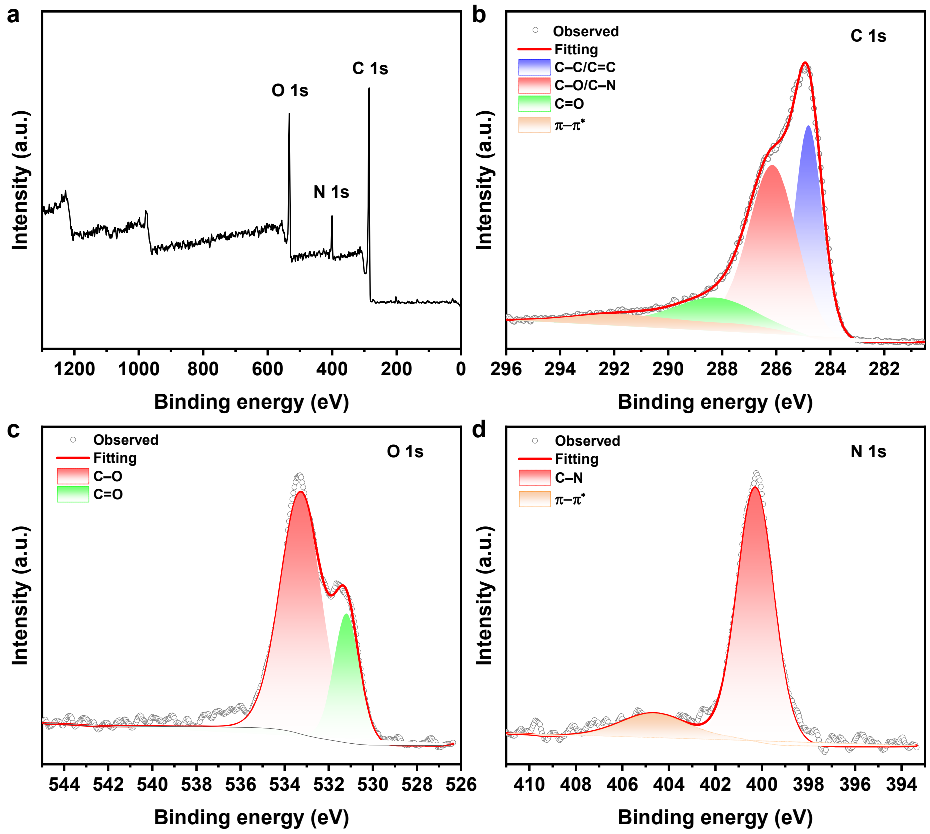

Figure S4. (**a**) Full scan XPS spectrum and (**b-d**) high-resolution XPS spectra of (b) C 1s, (c) O 1s, and (d) N 1s of Tp-BD$(OH)_2$ COF membrane.

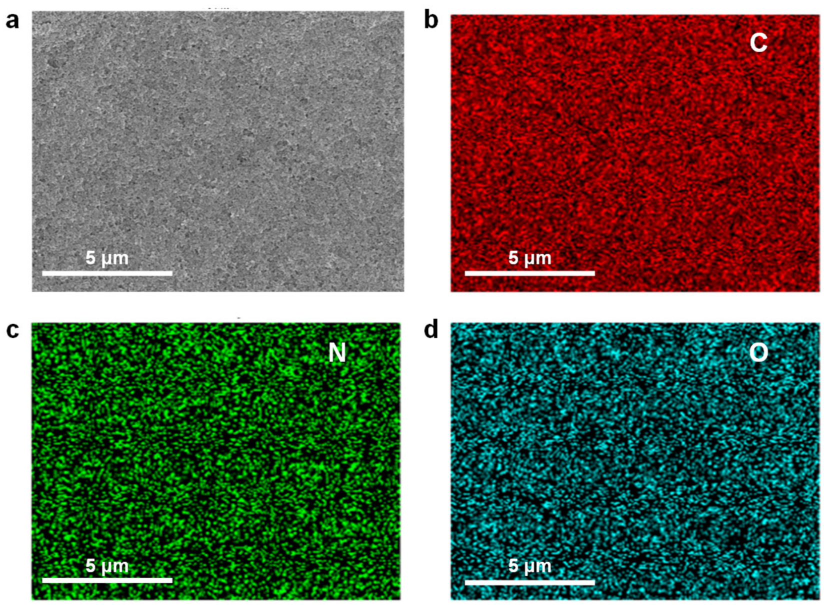

Figure S5. (**a**) Surface SEM image of Tp-BD$(OH)_2$ COF membrane. (**b-d**) Surface element distribution of (b) C, (c) N, and (d) O examined by energy dispersive X-ray spectroscopy (EDS).

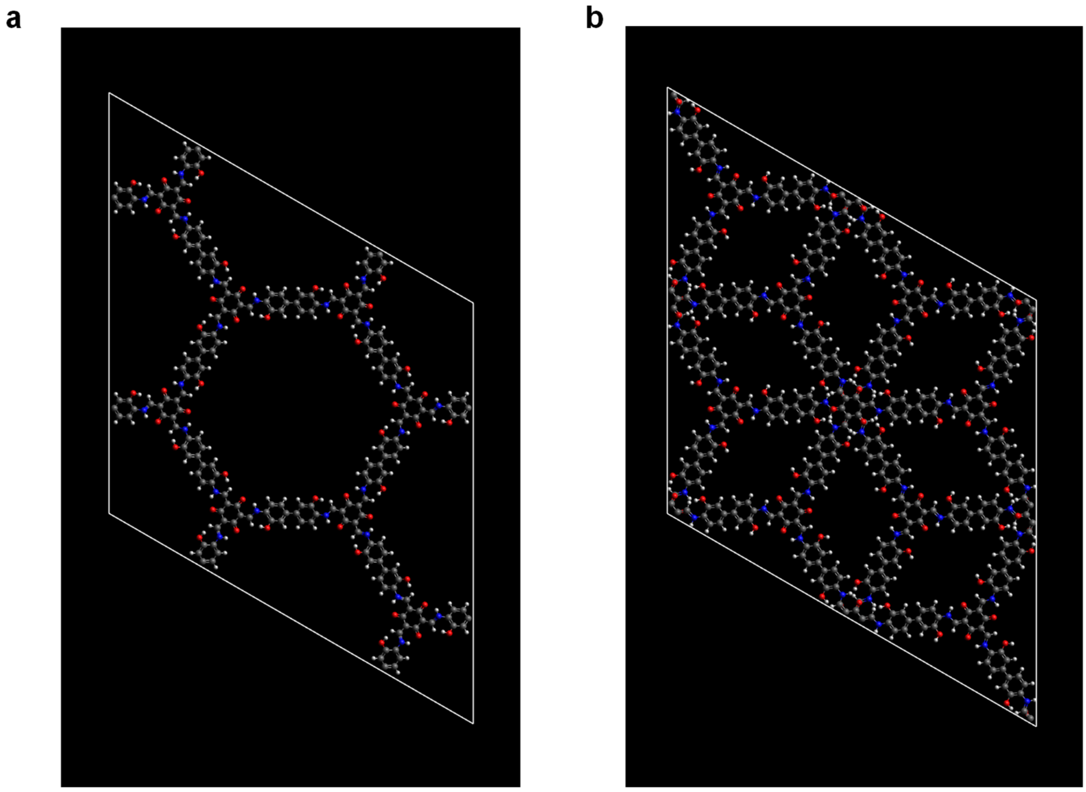

Figure S6. Theoretical schematic diagrams of (**a**) AA and (**b**) AB stacking modes of Tp-$BD(OH)_2$ COF membrane.

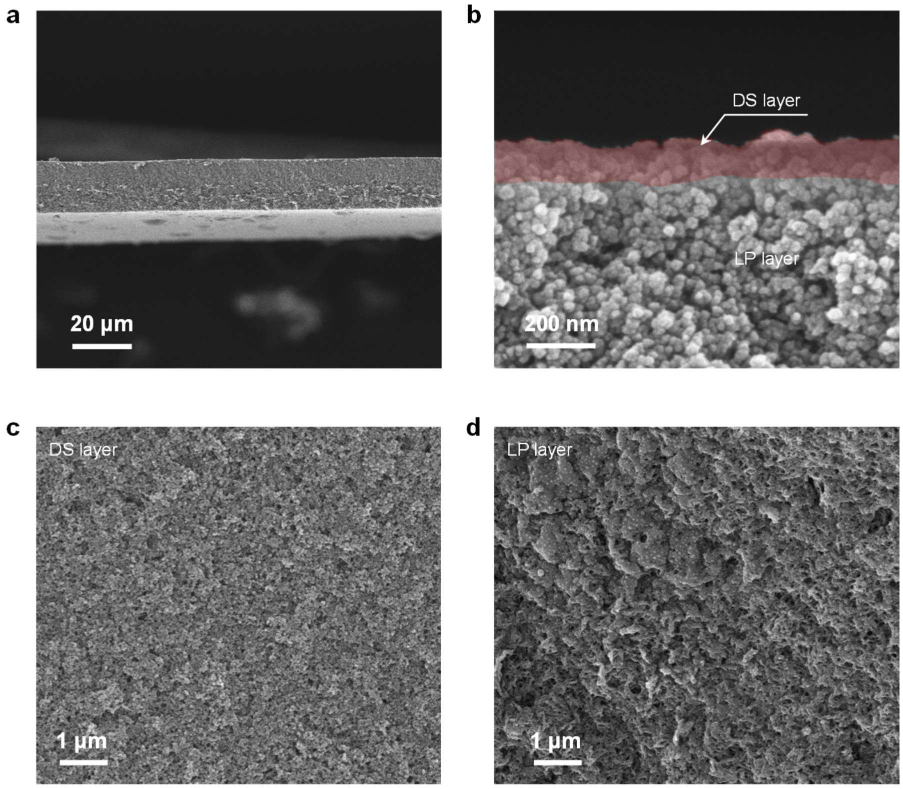

Figure S7. (**a-b**) Cross-section FESEM images of Tp-BD$(OH)_2$ COF membrane at (a) low magnification (1k ×) and (b) high magnification (100k ×). (**c-d**) FESEM images of the (c) DS layer side surface and (d) LP layer side surface of Tp-BD$(OH)_2$ COF membrane.

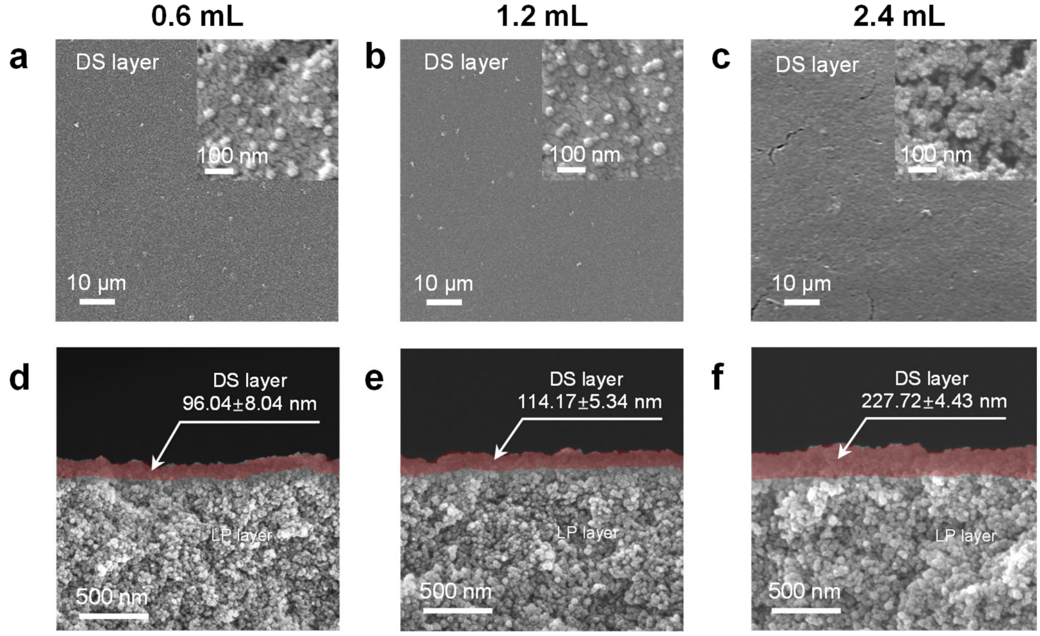

Figure S8. Effect of catalyst volume on the surface morphology and thickness of the DS layer of anisotropic Tp-BD$(OH)_2$ COF membrane. **(a-c)** FESEM images of the DS layer of the COF membrane prepared with a catalyst volume of (a) 0.6 mL, (b) 1.2 mL, and (c) 2.4 mL. The inset in the upper right corner of each image depicts the surface microstructure under high magnification. **(d-f)** Cross-section FESEM images of the corresponding DS layers.

Notes: The catalyst will significantly affect the morphology and thickness of the DS layer. Compared with the optimal condition (catalyst volume: 1.2 mL, Figure S8b and e), when the catalyst volume is only 0.6 mL, the overall surface of the DS layer is denser. However, there will be some small defects (Figure S8a), and its overall thickness is also thinner than the optimal condition (Figure S8d). When the catalyst dosage is 2.4 mL, cracks appear on the surface of the DS layer (Figure S8c), and its overall thickness is also thicker than the optimal condition (Figure S8f).

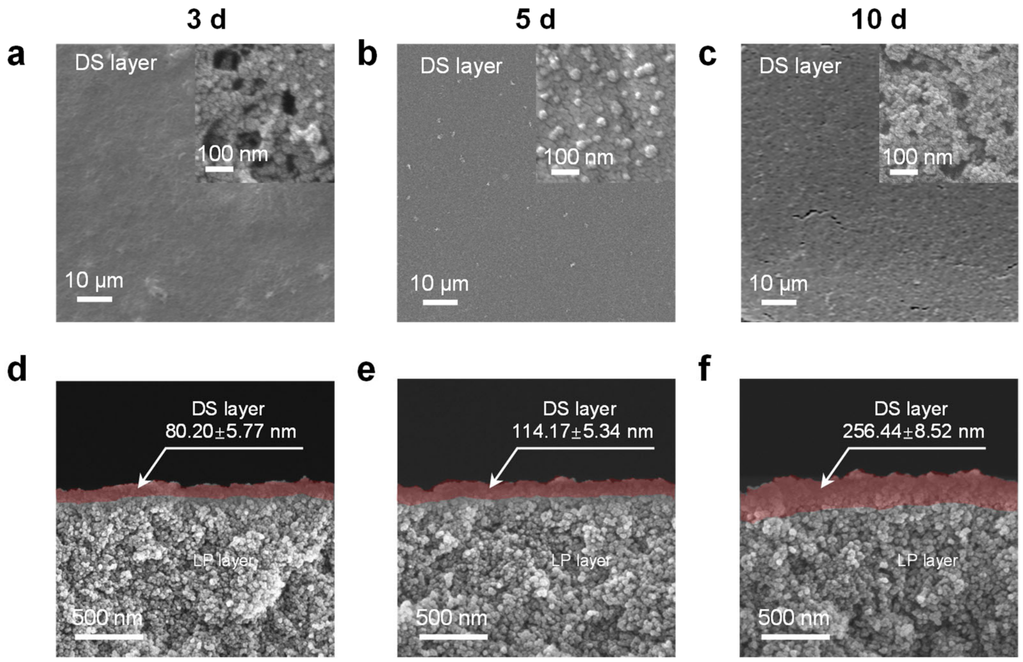

Figure S9. Effect of reaction time on the surface morphology and thickness of the DS layer of anisotropic Tp-BD(OH)$_2$ COF membrane. **(a-c)** FESEM images of the DS layer of the COF membrane prepared at a reaction time of (a) 3 d, (b) 5 d, and (c) 10 d. The inset in the upper right corner of each image depicts the surface microstructure under high magnification. **(d-f)** Cross-section FESEM images of the corresponding DS layers.

Notes: The reaction time will significantly affect the morphology and thickness of the DS layer. Compared with the optimal conditions (reaction time: 5 d, Figure S9b and e), when the reaction time is only 3 d, some small defects will appear on the surface of the DS layer. (Figure S9a), and its overall thickness is also thinner than the optimal condition (Figure S9d). When the reaction time is 10 d, cracks appear on the surface of the DS layer (Figure S9c), and its overall thickness is also thicker than the optimal condition (Figure S9f).

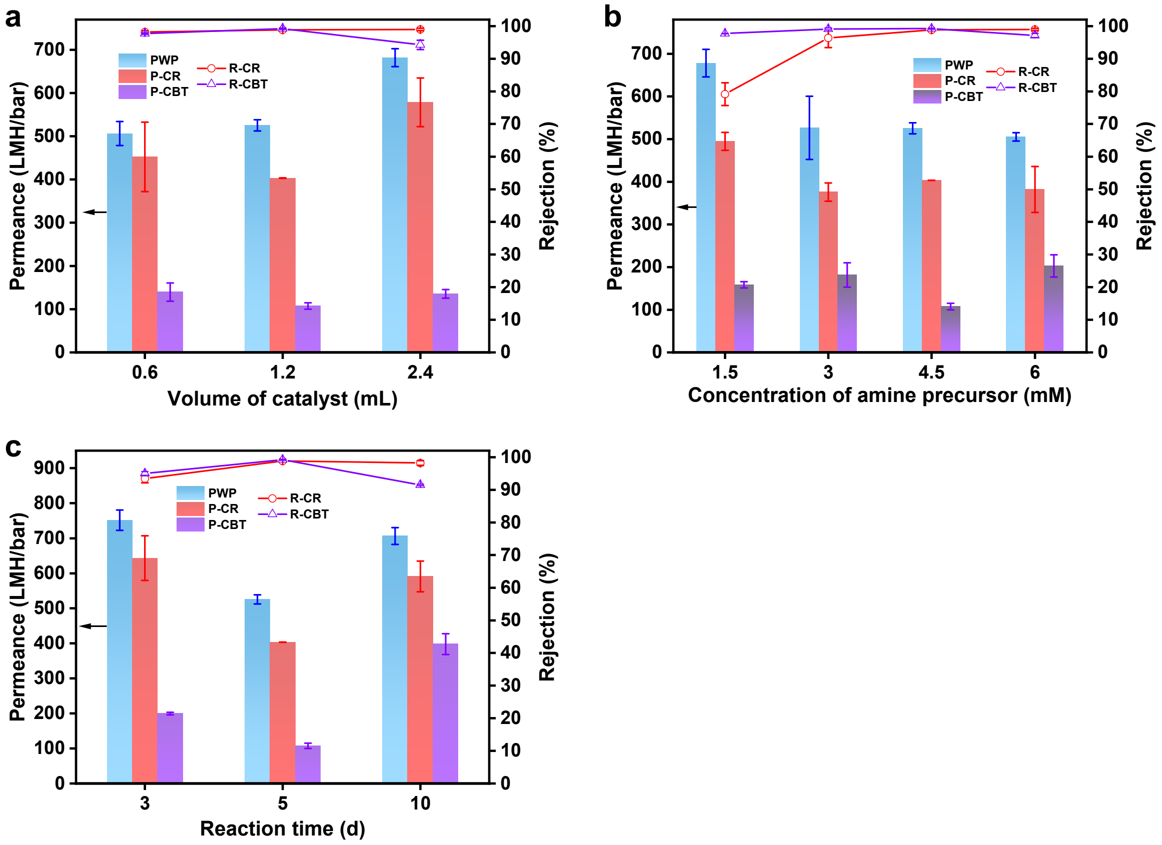

Figure S10. Optimization of synthesis conditions for the fabrication of Tp-BD$(OH)_2$ COF membrane**.** The influence of (**a**) volume of HAc catalyst, (**b**) concentration of BD$(OH)_2$ amine precursor, and (**c**) reaction time on membrane separation performance. (Feed: deionized water for pure water permeance (PWP) and 50.0 mg L^{-1} CR/CBT solutions for rejection tests, operation pressure: 1.5 bar). The Tp-BD$(OH)_2$ COF membrane prepared under the following conditions is the optimal one: 1.2 mL 3.0 mol L^{-1} HAc, 4.5 mM BD$(OH)_2$ amine precursor, 3.0 mM Tp aldehyde precursor, and the reaction time was 5 days.

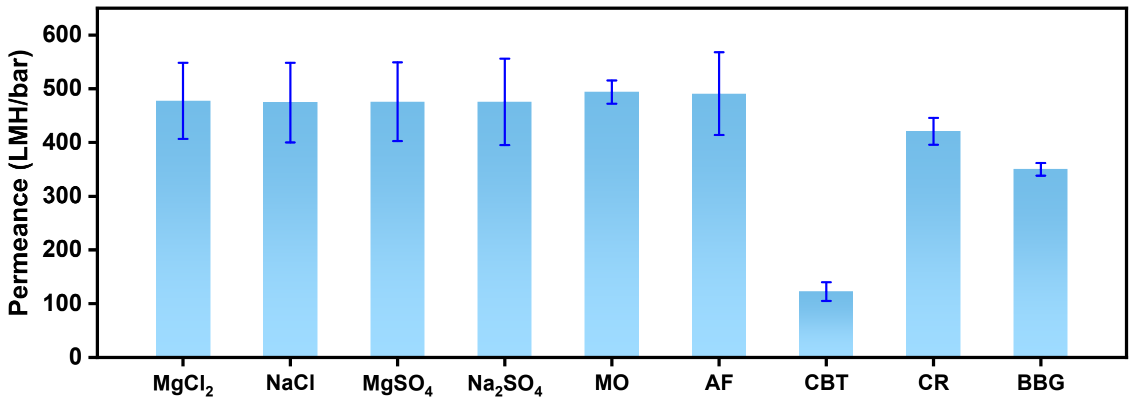

Figure S11. Water permeance of Tp-BD(OH)$_2$ COF membrane for filtrating different solutes.

(Salt concentration: 2.0 g L^{-1}, dye concentration: 50.0 mg L^{-1}, operation pressure: 1.5 bar)

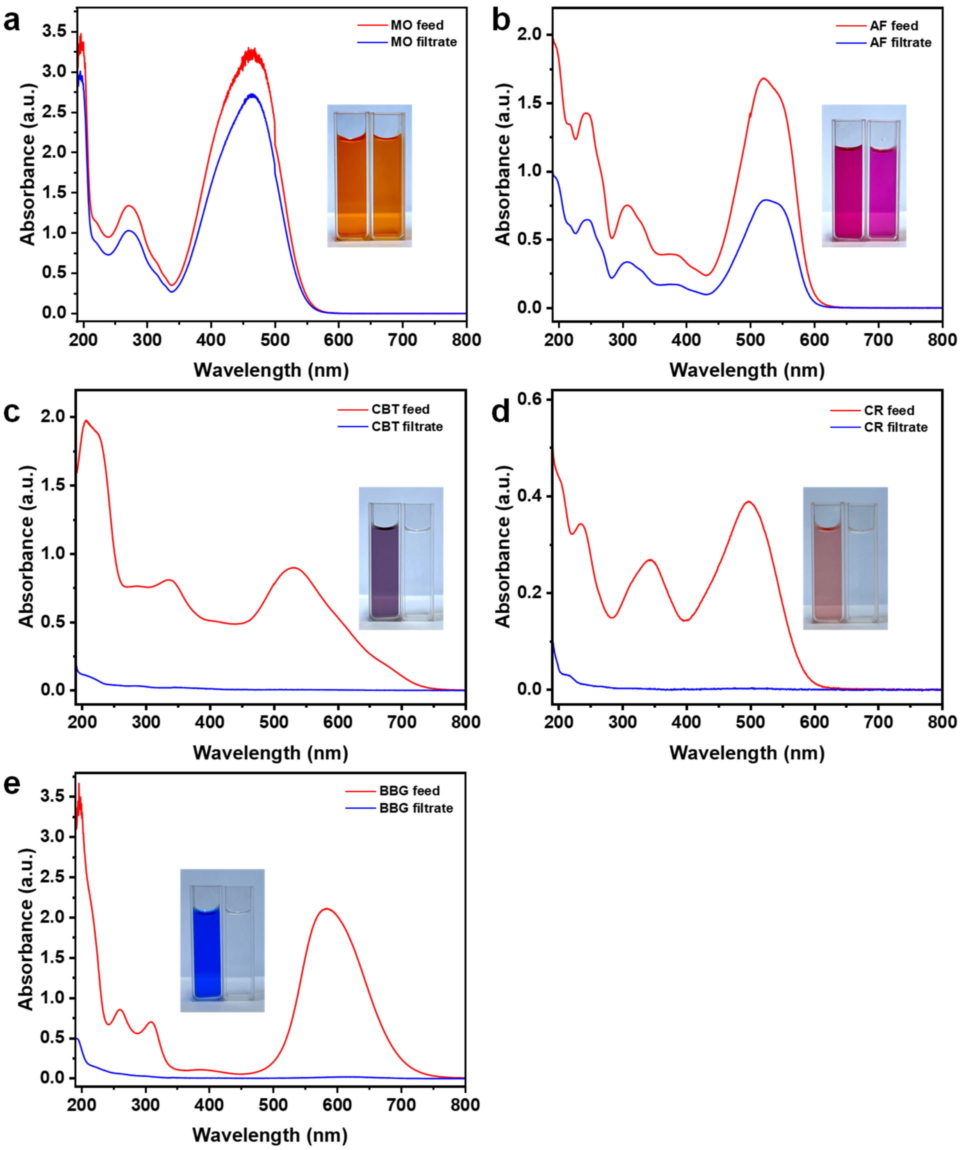

Figure S12. Dye separation performance of Tp-BD$(OH)_2$ COF membrane. UV-vis absorption spectra of (**a**) MO, (**b**) AF, (**c**) CBT, (**d**) CR, and (**e**) BBG dye molecules in the feed solution and filtrate. The inserted photograph in each figure visually demonstrates the color difference between the dye feed solution and the corresponding filtrate.

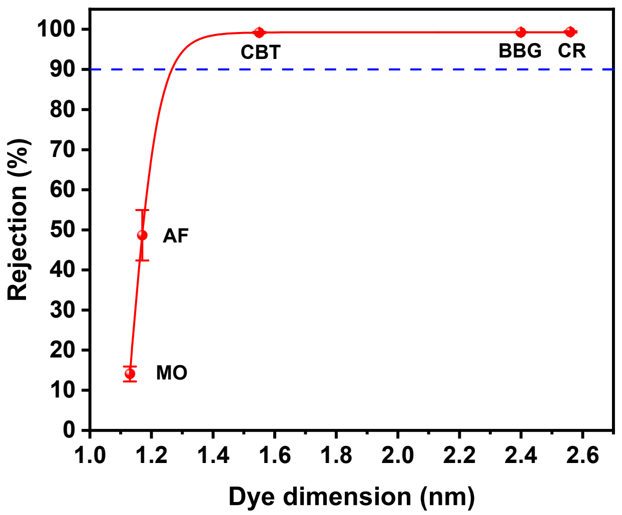

Figure S13. Rejections of dye molecules with different dimensions by Tp-BD$(OH)_2$ COF membrane. The longest side of each dye molecule was utilized to represent the dimension of the molecule (i.e., MO = 1.13 nm, AF = 1.17 nm, CBT = 1.55 nm, BBG = 2.40 nm, and CR = 2.56 nm).

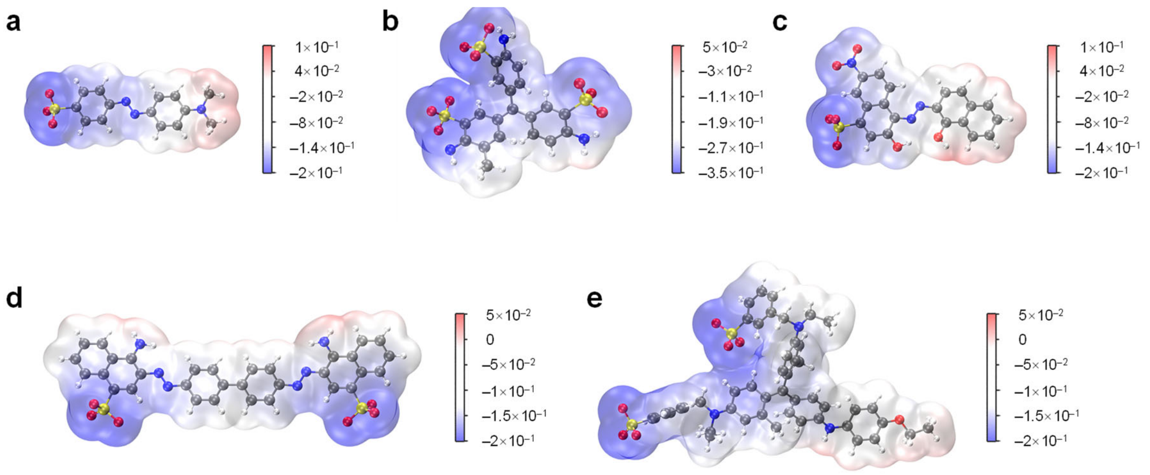

Figure S14. Visualization images of the electrostatic potential (ESP) distribution of (**a**) MO, (**b**) AF, (**c**) CBT, (**d**) CR, and (**e**) BBG molecules based on DFT calculations (scale bar unit: a.u.). The red, white, and blue colors in the visualization respectively represent the positive, neutral, and negative regions of the molecule.

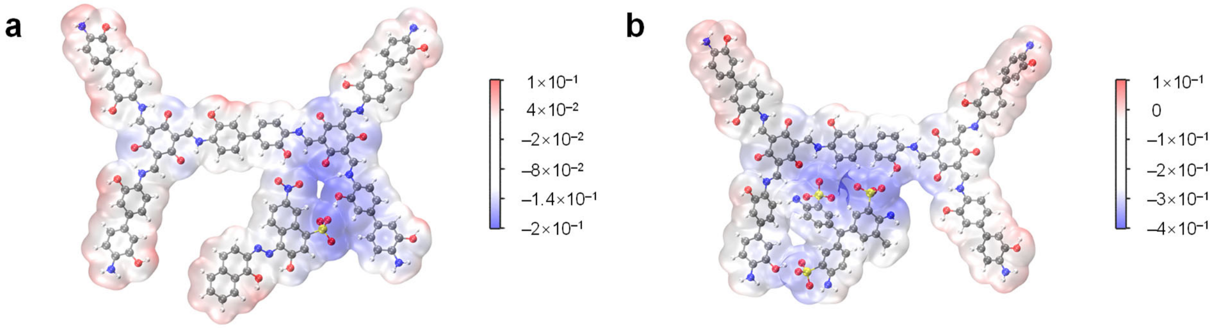

Figure S15. Visualization images of the electrostatic potential (ESP) distribution of (**a**) CBT/COF-fragment and (**b**) AF/COF-fragment configurations based on DFT calculations (scale bar unit: a.u.). The red, white, and blue colors in the visualization respectively represent the positive, neutral, and negative regions of the dye/COF-fragment configuration.

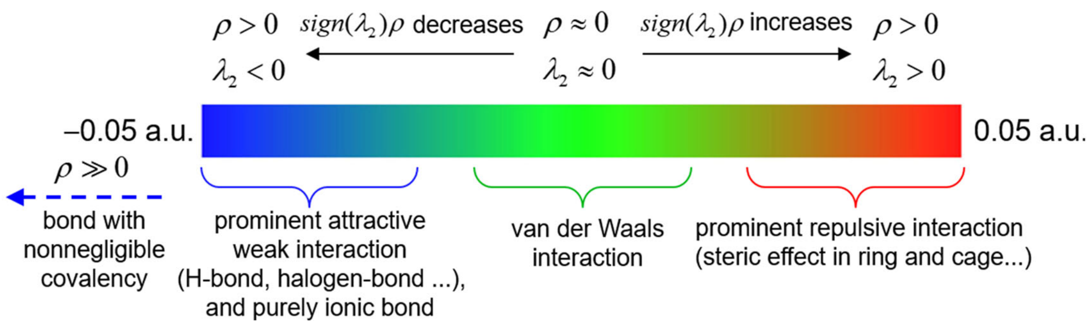

Figure S16. Interaction decomposition analysis of the binding configurations between COF molecular fragment and HAc, AF, and CBT. The blue, green, and red areas represent prominent attractive weak interaction, van der Waals interaction, and prominent repulsive interaction, respectively.

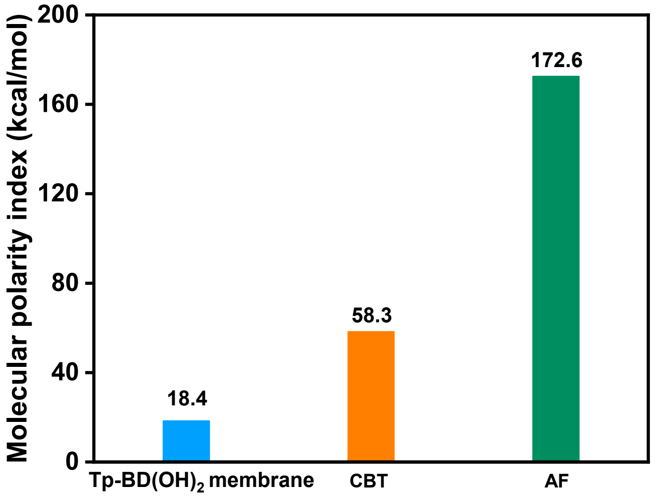

Figure S17. Molecular polarity index (MPI) of Tp-BD$(OH)_2$ COF membrane, CBT, and AF obtained by DFT calculations.

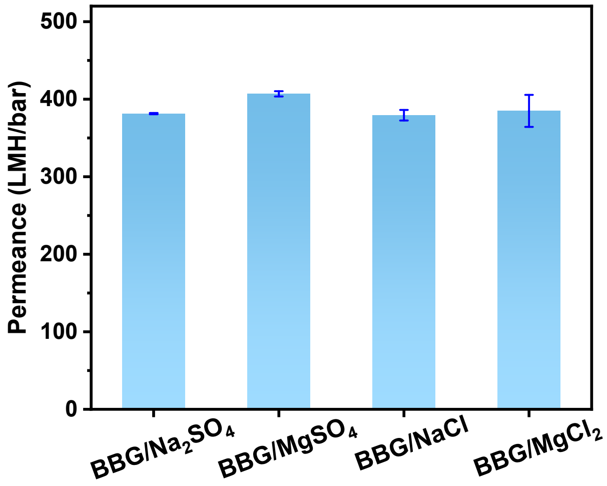

Figure S18. Water permeance of Tp-BD(OH)$_2$ COF membrane for filtrating the BBG/salts binary mixtures. (Salt concentration: 2.0 g L^{-1}, BBG concentration: 50.0 mg L^{-1}, operation pressure: 1.5 bar)

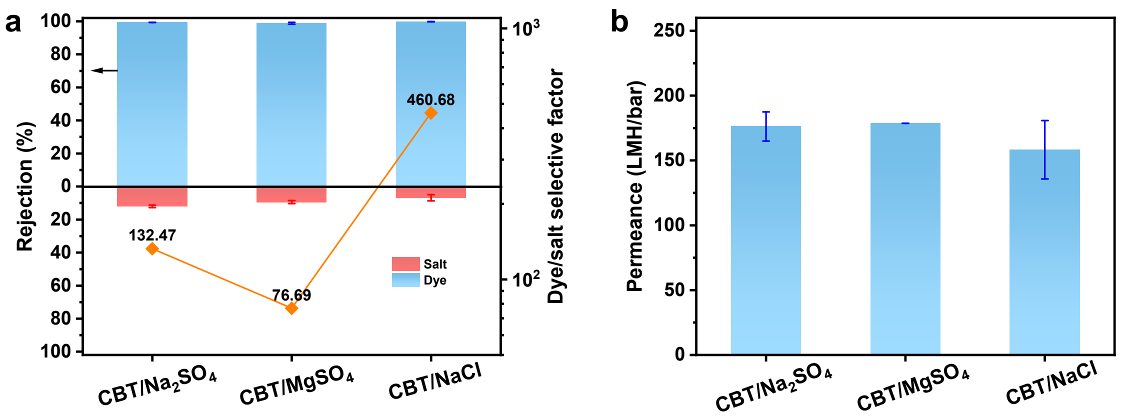

Figure S19. Separation performance of Tp-BD(OH)$_2$ COF membrane for filtrating the CBT/salts binary mixtures. (**a**) Rejection of CBT (blue column) and different salts (red column) and the corresponding CBT/salt selective factor (orange polyline). (**b**) Water permeance of Tp-BD(OH)$_2$ COF membrane for filtrating the CBT/salts binary mixtures. (Salt concentration: 2.0 g L^{-1}, CBT concentration: 50.0 mg L^{-1}, operation pressure: 1.5 bar)

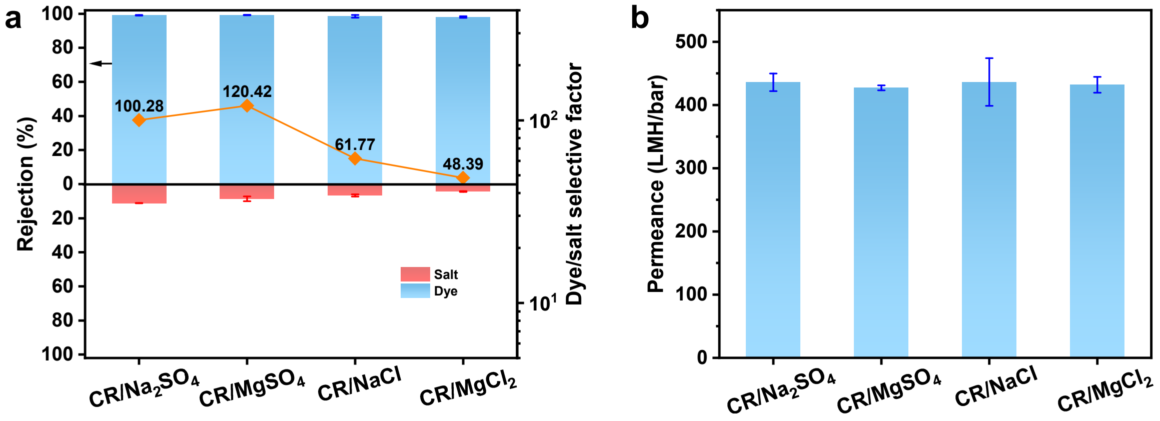

Figure S20. Separation performance of Tp-BD$(OH)_2$ COF membrane for filtrating the CR/salts binary mixtures. (**a**) Rejection of CR (blue column) and different salts (red column) and the corresponding CR/salt selective factor (orange polyline). (**b**) Water permeance of Tp-BD$(OH)_2$ COF membrane for filtrating the CR/salts binary mixtures. (Salt concentration: 2.0 g L^{-1}, CR concentration: 50.0 mg L^{-1}, operation pressure: 1.5 bar)

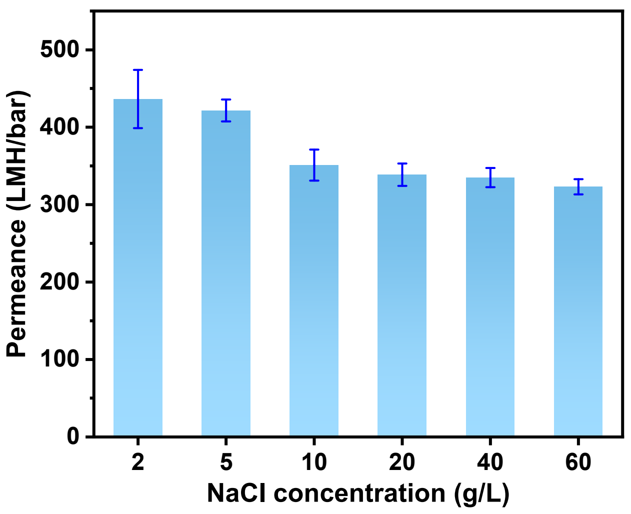

Figure S21. Water permeance of Tp-BD$(OH)_2$ COF membrane for filtrating the CR/NaCl binary mixtures with different NaCl concentrations. (CR concentration: 50.0 mg L^{-1}, NaCl concentration: 2.0–60.0 g L^{-1}, operation pressure: 1.5 bar)

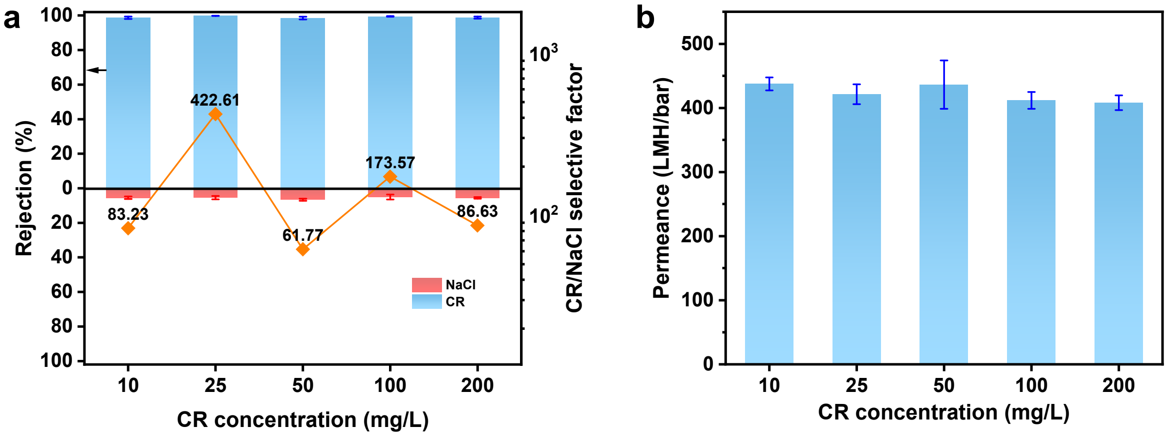

Figure S22. Separation performance of Tp-BD$(OH)_2$ COF membrane for filtrating the CR/NaCl binary mixtures with different CR concentrations. (**a**) Rejection of CR (blue column) and NaCl (red column) and the corresponding CR/NaCl selective factor (orange polyline). (**b**) Water permeance of Tp-BD$(OH)_2$ COF membrane for filtrating the CR/NaCl binary mixtures. (NaCl concentration: 2.0 g L^{-1}, CR concentration: 10.0–200.0 mg L^{-1}, operation pressure: 1.5 bar)

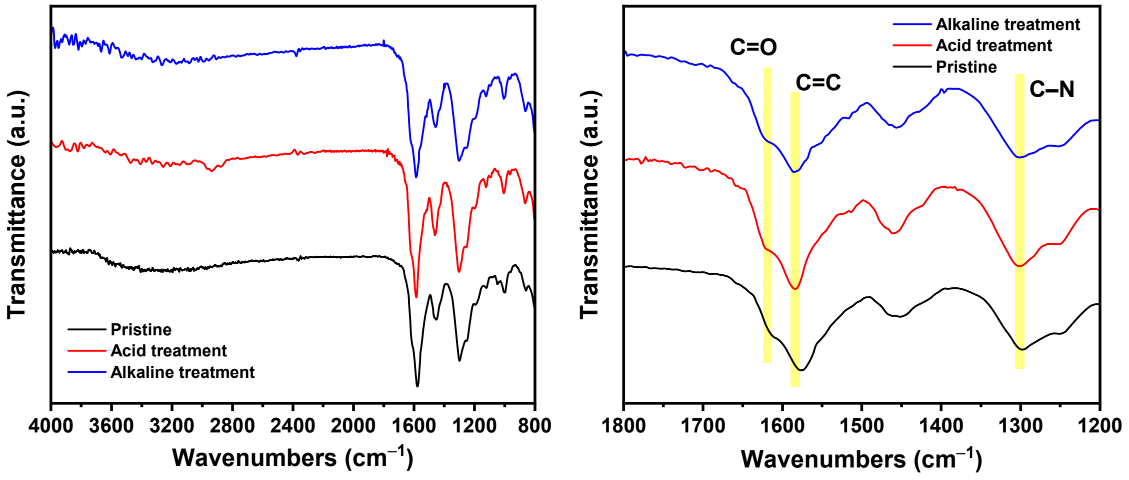

Figure S23. FT-IR spectra of the Tp-BD(OH)$_2$ COF membrane before and after treatment with strong acid or alkali.

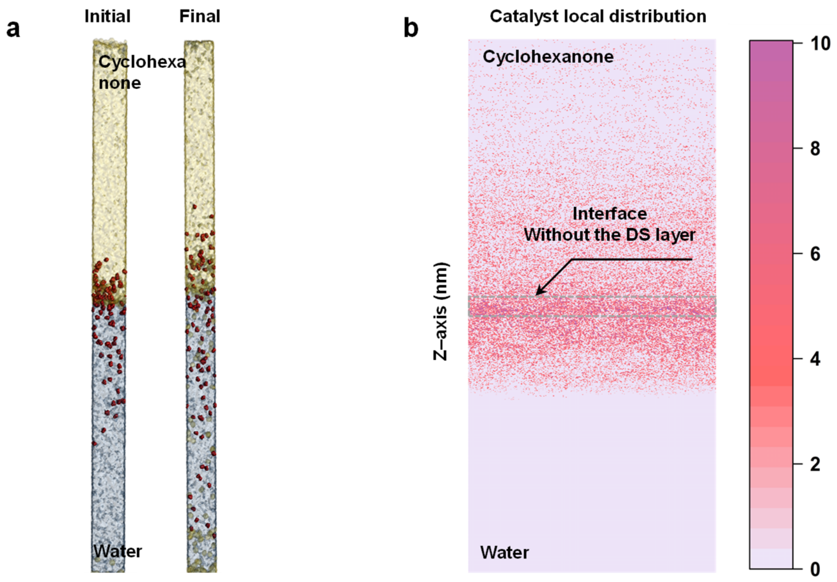

Figure S24. (**a**) Molecular dynamics (MD) simulation snapshots of HAc diffusion from the aqueous phase across the interface into the cyclohexanone phase at 0 and 17000 ps in a system without a COF dense selective (DS) layer at the interface. (**b**) The obtained average density distribution of HAc along the Z-axis (frontal view) simulated within 20 ns for the system without a dense COF layer at the interface (scale bar unit: counts).

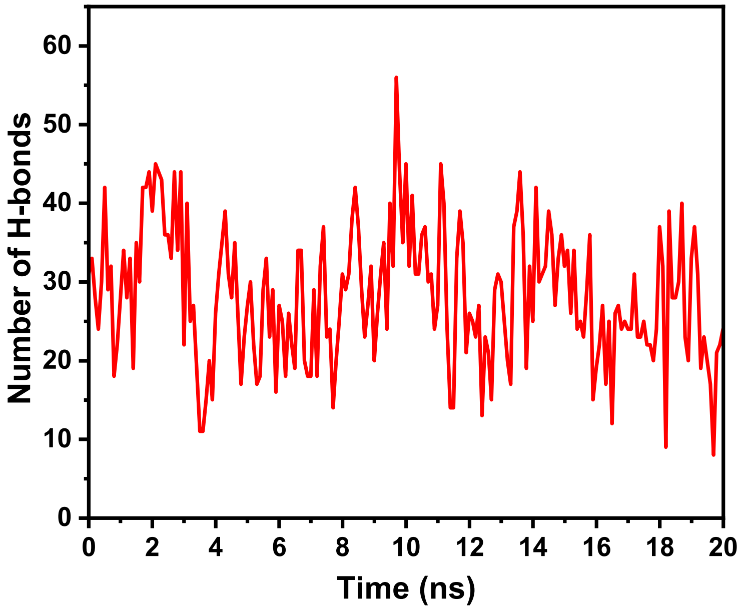

Figure S25. The number of H-bonds formed between the Tp-BD$(OH)_2$ COF structure and acetic acid (HAc) during membrane formation obtained from MD simulations.

3. Supplementary Tables

Table S1. Semiquantitative analysis of the C, N, and O elemental contents in Tp-BD$(OH)_2$ COF membrane based on the XPS data.

	**C atomic %**	**N atomic %**	**O atomic %**
**Exp.**	72.56	8.95	18.49
**Anal. Calcd.**	68.39	10.88	20.73

Table S2. Fractional atomic coordinates for the AA stacking mode of Tp-BD(OH)$_2$ COF membrane.

**AA stacking**			
**Hexagonal, P6**			
**a=b=30.30 Å, c=3.49 Å**			
**α=β=90.00°, γ=120.00°**			
**Atom list**	**x**	**y**	**z**
C1	0.5165	0.48842	0.0467
C2	0.49509	0.43531	0.0467
C3	0.52591	0.4131	0.0467
C4	0.57951	0.44441	0.0467
C5	0.60078	0.49733	0.0467
C6	0.56971	0.51919	0.0467
N7	0.6137	0.4246	0.0467
O8	0.50298	0.36111	0.0467
C9	0.6879	0.38919	0.0467
C10	0.63172	0.35454	0.0467
O11	0.27042	0.70576	0.0467
C12	1.22683	0.59859	0.0467
H13	0.45191	0.40954	0.0467
H14	0.64395	0.52315	0.0467
H15	0.58796	0.56246	0.0467

H16	0.65551	0.45107	0.0467
H17	0.46399	0.34456	0.16751
H18	1.21477	0.55641	0.0467

Table S3. Fractional atomic coordinates for the AB stacking mode of Tp-BD$(OH)_2$ COF membrane.

AB stacking<br>Trigonal, P-3<br>a=b=30.33 Å, c=5.00 Å<br>α=β=90.00°, γ=120.00°			
**Atom list**	**x**	**y**	**z**
C1	0.36141	0.17843	-0.23386
C2	0.38419	0.14804	-0.23386
C3	0.43708	0.17025	-0.23386
C4	0.46845	0.22316	-0.23386
C5	0.44576	0.25414	-0.23386
C6	0.39274	0.23152	-0.23386
N7	0.52243	0.24302	-0.23386
O8	0.4754	0.30827	-0.23386
C9	0.61063	0.31254	-0.23386
C10	0.64584	0.36825	-0.23386
C11	0.56041	0.2957	-0.23386
O12	0.62831	0.39678	-0.23386
C13	1.30526	0.15491	-0.23386
C14	1.28248	0.18529	-0.23386
C15	1.22958	0.16308	-0.23386

C16	1.19822	0.11017	-0.23386
C17	1.22091	0.07919	-0.23386
C18	1.27392	0.10181	-0.23386
N19	1.14424	0.09032	-0.23386
O20	1.19127	0.02507	-0.23386
C21	1.05604	0.0208	-0.23386
C22	1.02082	-0.03491	-0.23386
C23	1.10626	0.03763	-0.23386
O24	1.03835	-0.06345	-0.23386
H25	0.35947	0.10478	-0.23386
H26	0.45523	0.14518	-0.23386
H27	0.37505	0.257	-0.23386
H28	0.5378	0.21661	-0.23386
H29	0.45383	0.32425	-0.13345
H30	0.54867	0.32602	-0.23386
H31	1.3072	0.22855	-0.23386
H32	1.21143	0.18815	-0.23386
H33	1.29161	0.07633	-0.23386
H34	1.12888	0.11673	-0.23386
H35	1.1543	0.01293	-0.13344
H36	1.118	0.00731	-0.23386

Table S4. Pure water permeance comparison of the Tp-BD(OH)$_2$ COF membrane with the recently reported COF membranes.

Number	COF membrane	Pure water permeance (LMH bar^{-1})	Ref.
1	FS-COM	10.0	[8]
2	TFPM-HZ/PAN	10.0	[9]
3	sTPA-PZ	13.1	[10]
4	Tp-TTA/mPSFx	36.5	[11]
5	TpTG$_{Cl}$@CNFs	42.8	[12]
6	TpPa-SO$_3$H	42.9	[13]
7	TAPB-DHTA	48.0	[14]
8	pDA/TpPa(W/E)-COF	53.1	[15]
9	TpBdMe/PSf	62.2	[16]
10	QL-COFs	85.0	[17]
11	TpBd/PSf	85.0	[18]
12	PANI-TpPa	85.2	[19]
13	TAPA-TFP	148.2	[20]
14	TpPa2Cl/Alumina	150.0	[21]
15	Tp-Bpy	211.0	[22]
	**Tp-BD(OH)$_2$**	**525.3**	**This work**

Table S5. Summary of the molecular properties of the dyes used in this study.

**Dyes**	**Molecular weight ($g \cdot mol^{-1}$)**	**Molecular size [11, 13, 23] (nm × nm)**	**Electrical properties**	**λ_{max} (nm)**
MO	327.33	1.13 × 0.42	-	462
AF	585.54	1.17 × 1.13	-	520
CBT	461.38	1.55 × 0.88	-	530
CR	696.67	2.56 × 0.73	-	500
BBG	854.04	2.40 × 1.50	-	582

Table S6. Various molecular configurations and the corresponding single point energies (SPE).

Name	Molecular configuration	SPE (Hartree)
COF fragment	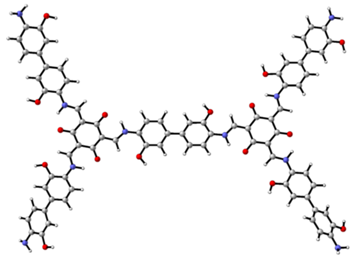	−4759.326858
CBT	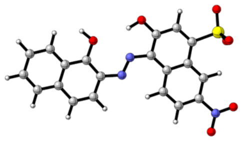	−1858.300471
AF	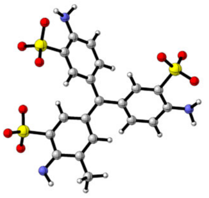	−2807.949633
AF/COF fragment	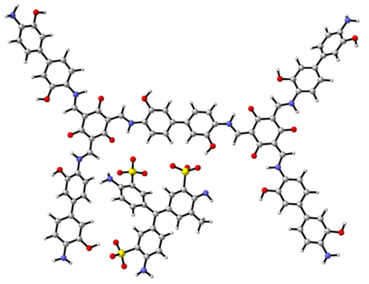	−7567.306163
CBT/COF fragment	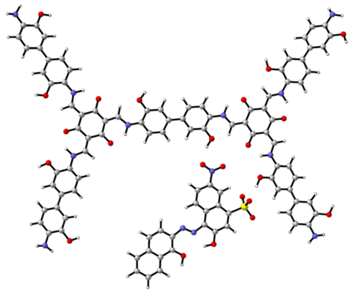	−6617.647626

Table S7. Adsorption rates of three dye molecules by the Tp-BD$(OH)_2$ COF membrane.

Dyes	Adsorbed rate (%)
CBT	4.61
CR	2.86
BBG	6.02

Table S8. Molecular sieving performance summary of the Tp-BD$(OH)_2$ COF membrane and other state-of-the-art NF membranes reported in the literature.

Membrane	Water permeance (LMH bar^{-1})	Dyes	Dye rejections (%)	Salts	Salt rejections (%)	Selective factor of dye/NaCl	Ref.
COF-LZU1 PVP/PVDF	19.37	Congo Red	96.30	Na_2SO_4	4.50	26.59	[24]
		Eriochrome Black T	94.50	$MgSO_4$	3.50	17.89	
		Methylene Blue	96.10	NaCl	1.60	25.23	
		Acid Red	94.50	$MgCl_2$	2.80	17.89	
COF-LZU1	76	Methyl Blue	99.20	Na_2SO_4	3.20	119.38	[25]
		Congo Red	98.60	NaCl	4.50	68.21	
		Chrome Black T	98.20	$MgCl_2$	5.40	53.06	
		Rose Bengal	99.10	$CaCl_2$	4.70	106.11	
		Acid Fuchsin	91.40	$AlCl_3$	8.70	11.10	

		Rhodamine B	84.50	KCl	3.00	6.16	
TpDMTH-2	109.73	Congo Red	99.63	Na_2SO_4	3.03	267.81	[26]
		Methyl Blue	98.64	$MgSO_4$	2.42	72.86	
		Coomassie brilliant Blue	99.51	NaCl	0.91	202.22	
		Acid Orange II	41.30	$MgCl_2$	1.78	1.69	
MXene/COFs	200.2	Congo Red	99.10	Na_2SO_4	10.00	109.89	[27]
		Eriochrome Black T	98.90	$MgSO_4$	4.90	89.91	
		Methyl Blue	98.70	NaCl	1.10	76.08	
		Direct Red 80	99.20	$MgCl_2$	3.10	123.63	
		Direct Black 38	99.10			109.89	
		Rhodamine B	95.80			23.55	
MPD-TFB	94.4	Coomassie Brilliant Blue	99.50	Na_2SO_4	8.10	194.20	[28]

		Congo Red	99.40	$MgSO_4$	7.40	161.83	
		Basic Green 4	99.50	NaCl	2.90	194.20	
		Rhodamine B	99.50	$MgCl_2$	4.90	194.20	
CTF-MMM-0.1	204	Reactive Black 5	98.53	Na_2SO_4	26.14	61.86	[29]
		Rhodamine B	50.00	$MgSO_4$	8.65	1.82	
		Eriochrome Black T	62.00	NaCl	9.07	2.39	
		Methyl Blue	90.00	$MgCl_2$	13.32	9.09	
		Congo Red	80.00			4.55	
iCOF-3	68	Methyl orange	28.00	Na_2SO_4	3.23	1.35	[30]
		Rose Bengal	97.00	$MgSO_4$	5.13	32.51	
				NaCl	2.48		
				$MgCl_2$	5.07		
Cu-TpPa-HPAN	89.8	Congo Red	99.10	Na_2SO_4	41.00	104.44	[31]
		Eriochrome Black T	99.00	$MgSO_4$	8.00	94.00	

		Acid Fuchsin	78.60	NaCl	6.00	4.39	
		Lemon Yellow	94.80	$MgCl_2$	3.00	18.08	
		Acid Orange 7	84.60			6.10	
GO/COF-1	31	Congo Red	99.08	Na_2SO_4	4.46	101.59	[32]
		Methylene Blue	98.46	$MgSO_4$	7.88	60.69	
		Reactive Black 5	90.19	NaCl	6.54	9.53	
		Direct Red	97.00	$MgCl_2$	11.30	31.15	
		Eriochrome Black T	100.00			/	
BIC@MIL-101(Fe)	43.2	Eriochrome Black T	98.40	Na_2SO_4	4.80	61.19	[33]
		Congo Red	99.70	$MgSO_4$	4.90	326.33	
		Chlorazol Black T	99.80	NaCl	2.10	489.50	
				$MgCl_2$	2.90		
LNM-3	24.5	Methyl Blue	99.18	Na_2SO_4	17.50	112.80	[34]
				NaCl	7.50		

AM-PEI/HPAN	42.9	Methyl Blue	99.20	Na_2SO_4	36.30	119.50	[35]
		Congo Red	98.60	$MgSO_4$	15.60	68.29	
		Crystal Violet	75.80	NaCl	4.40	3.95	
				$MgCl_2$	17.70		
LNFM-2	53.23	Congo Red	99.62	Na_2SO_4	11.00	248.42	[36]
		Direct Red	95.22	NaCl	5.60	19.75	
		Reactive Blue 2	99.55			209.78	
TFC_{ERT}	29.18	Congo Red	99.75	Na_2SO_4	14.05	394.64	[37]
				NaCl	1.34		
POP/TMC	31.28	Reactive Black 5	99.13	Na_2SO_4	25.53	104.60	[38]
		Reactive Red 49	95.17	NaCl	9.00	18.84	
Su0.6/TMC0.1	52.4	Congo Red	99.40	Na_2SO_4	11.20	161.17	[39]
		Direct Red	98.80	NaCl	3.30	80.58	
		Reactive Blue 2	98.20			53.72	

$SaTG_{Cl}$/TMC	97.2	Eriochrome Black T	99.40	Na_2SO_4<br>NaCl	15.80<br>2.30	162.83	[40]
resveratrol/TMC	121.1	Congo Red	99.40	Na_2SO_4<br>NaCl	9.80<br>4.20	159.67	[41]
PVDF/CD-0.75/25	18.8	Congo Red<br>Coomassie Brilliant Blue	99.00<br>99.20	Na_2SO_4<br>NaCl	9.90<br>6.80	93.20<br>116.50	[42]
EGCG-based LNF-4	41.7	Congo Red<br>Methyl Blue<br>Evans Blue	98.50<br>93.40<br>94.80	Na_2SO_4<br>NaCl	37.30<br>7.40	61.73<br>14.03<br>17.81	[43]
TAIP membrane (M4)	32.57	Congo Red<br>Rose Bengal	99.40<br>99.19	Na_2SO_4<br>NaCl	9.41<br>2.25	162.92<br>120.68	[44]
Arg/TMC	130.4	Congo Red<br>Direct Red 23	99.60<br>99.00	Na_2SO_4<br>NaCl	7.20<br>3.80	240.50<br>96.20	[45]

		Reactive Blue 2	98.50			64.13	
		Reactive Orange 16	95.20			20.04	
TFC-3	43.8	Congo Red	99.20	Na_2SO_4	~25.00	103.13	[46]
		Coomassie Blue G-250	99.70	NaCl	17.50	275.00	
		Reactive Blue 19	99.70			275.00	
		Methyl Blue	98.80			68.75	
PEA-TMC	16.6	Methyl Blue	95.40	Na_2SO_4	29.40	18.17	[47]
				$MgSO_4$	19.20		
				NaCl	16.40		
				$MgCl_2$	11.10		
Tp-BD(OH)$_2$	525.33	Eriochrome Black T	99.80	NaCl	6.80	**460.68**	**This work**
		Congo Red	99.77	NaCl	4.16	**416.70**	
		Brilliant Blue G	99.86	NaCl	6.57	**648.59**	

References

[1] M. J. Abraham, T. Murtola, R. Schulz, S. Páll, J. C. Smith, B. Hess, E. Lindahl, *SoftwareX* **2015**, *1-2*, 19.

[2] F. Neese, *WIREs Comput. Mol. Sci.* **2022**, *12*, e1606.

[3] a) A. D. Becke, *J. Chem. Phys.* **1992**, *96*, 2155; b) C. Lee, W. Yang, R. G. Parr, *Phys. Rev. B.* **1988**, *37*, 785; c) S. Grimme, S. Ehrlich, L. Goerigk, *J. Comput. Chem.* **2011**, *32*, 1456; d) S. Grimme, J. Antony, S. Ehrlich, H. Krieg, *J. Chem. Phys.* **2010**, *132*.

[4] a) S. Manzetti, T. Lu, *J. Phys. Org. Chem.* **2013**, *26*, 473; b) T. Lu, S. Manzetti, *Struct. Chem.* **2014**, *25*, 1521; c) T. Lu, F. Chen, *J. Comput. Chem.* **2012**, *33*, 580.

[5] a) F. Weigend, R. Ahlrichs, *Phys. Chem. Chem. Phys.* **2005**, *7*, 3297; b) G. L. Stoychev, A. A. Auer, F. Neese, *J. Chem. Theory. Comput.* **2017**, *13*, 554.

[6] A. V. Marenich, C. J. Cramer, D. G. Truhlar, *J. Phys. Chem. B* **2009**, *113*, 6378.

[7] T. Lu, Q. Chen, *J. Comput. Chem.* **2022**, *43*, 539.

[8] Y. Li, Q. Wu, X. Guo, M. Zhang, B. Chen, G. Wei, X. Li, X. Li, S. Li, L. Ma, *Nat. Commun.* **2020**, *11*, 599.

[9] X. Shi, Z. Zhang, C. Yin, X. Zhang, J. Long, Z. Zhang, Y. Wang, *Angew. Chem. Int. Ed.* **2022**, *61*, e202207559.

[10] R. Guo, Z. Zha, J. Wang, Z. Wang, M. D. Guiver, S. Zhao, *Small* **2024**, *20*, 2308904.

[11] Y. Zhang, H. Ye, D. Chen, N. Li, Q. Xu, H. Li, J. He, J. Lu, *J. Membr. Sci.* **2021**, *628*, 119216.

[12] H. Yang, L. Yang, H. Wang, Z. Xu, Y. Zhao, Y. Luo, N. Nasir, Y. Song, H. Wu, F. Pan, Z. Jiang, *Nat. Commun.* **2019**, *10*, 2101.

[13] X. Liu, J. Wang, Y. Shang, C. T. Yavuz, N. M. Khashab, *J. Am. Chem. Soc.* **2024**, *146*, 2313.

[14] K. Liu, C. Yin, J. Gao, Y. Wang, *Angew. Chem. Int. Ed.* **2024**, *n/a*, e202422333.

[15] Y. Zhang, J. Guo, G. Han, Y. Bai, Q. Ge, J. Ma, C. H. Lau, L. Shao, *Sci. Adv.* **2021**, *7*, eabe8706.

[16] T. Wang, H. Wu, S. Zhao, W. Zhang, M. Tahir, Z. Wang, J. Wang, *Chem. Eng. J.* **2020**, *384*, 123347.

[17] Y. Yang, L. Yu, T. Chu, H. Niu, J. Wang, Y. Cai, *Nat. Commun.* **2022**, *13*, 2615.

[18] R. Lei, Z. Zha, Z. Hao, J. Wang, Z. Wang, S. Zhao, *J. Membr. Sci.* **2022**, *650*, 120431.

[19] C. Mao, S. Zhao, P. He, Z. Wang, J. Wang, *Chem. Eng. J.* **2021**, *414*, 128929.

[20] J. Liu, G. Han, D. Zhao, K. Lu, J. Gao, T.-S. Chung, *Sci. Adv.* **2020**, *6*, eabb1110.

[21] W. J. S. Siow, J. Y. Chong, J. H. Ong, M. Kraft, R. Wang, R. Xu, *Angew. Chem. Int. Ed.* **2024**, *63*, e202406830.

[22] K. Dey, M. Pal, K. C. Rout, S. Kunjattu H, A. Das, R. Mukherjee, U. K. Kharul, R. Banerjee, *J. Am. Chem. Soc.* **2017**, *139*, 13083.

[23] L.-Y. Chen, Y.-N. Gai, X.-T. Gai, J. Qin, Z.-G. Wang, L.-S. Cui, H. Guo, M.-Y. Jiang, Q. Zou, T. Zhou, J.-G. Gai, *Chem. Eng. J.* **2022**, *430*, 133024.

[24] Y. Pan, Z. Li, S. Shen, D. Liu, G. Zhang, *Colloids Surf. Physicochem. Eng. Aspects* **2024**, *686*, 133431.

[25] H. Fan, J. Gu, H. Meng, A. Knebel, J. Caro, *Angew. Chem. Int. Ed.* **2018**, *57*, 4083.

[26] J.-Y. Dai, Y.-X. Fang, Z.-L. Xu, D. Pandaya, J. Liang, H.-F. Yan, Y.-J. Tang, *Desalination* **2023**, *568*, 117025.

[27] Y. Q. Zhang, S. W. Tian, Q. K. Sha, K. L. Ding, X. H. Yan, X. X. Zhang, N. Han, *J. Membr. Sci.* **2024**, *704*, 122857.

[28] Y.-X. Fang, Y.-F. Lin, Z.-L. Xu, J.-W. Mo, P.-P. Li, *J. Membr. Sci.* **2023**, *673*, 121470.

[29] L. Zhang, J. Wang, Y. Zhang, L. Zang, Y. Yang, Y. Zhang, X. Wang, L. Sun, *Sep. Purif. Technol.* **2024**, *348*, 127745.

[30] Q. Miao, Y. Wang, D. Chen, N. Cao, J. Pang, *J. Hazard. Mater.* **2024**, *465*, 133049.

[31] Y. Zhang, S. Tian, Q. Sha, T. Yang, Y. Huang, X. Zhang, W. Wang, N. Han, *J. Membr. Sci.* **2024**, 122907.

[32] X. Zhang, H. Li, J. Wang, D. Peng, J. Liu, Y. Zhang, *J. Membr. Sci.* **2019**, *581*, 321.

[33] Y.-H. Tong, Y.-Z. Wu, Z.-L. Xu, L.-H. Luo, S.-J. Xu, *Chem. Eng. J.* **2022**, *444*, 136507.

[34] J. Wang, R. He, X. Han, D. Jiao, J. Zhu, F. Lai, X. Liu, J. Liu, Y. Zhang, B. Van der Bruggen, *Chem. Eng. J.* **2019**, *375*, 121982.

[35] L. Zhang, L. Xu, H. Yu, P. Yao, M. Zhang, F. Guo, L. Yu, *J. Membr. Sci.* **2022**, *641*, 119923.

[36] P. Jin, J. Zhu, S. Yuan, G. Zhang, A. Volodine, M. Tian, J. Wang, P. Luis, B. Van der Bruggen, *Chem. Eng. J.* **2021**, *406*, 126796.

[37] M. B. M. Y. Ang, G.-W. Huang, M.-Y. Chu, J. C. Millare, S.-H. Huang, K.-R. Lee, *J. Water Process. Eng.* **2022**, *48*, 102843.

[38] R. Li, Z. Mai, D. Peng, S. Xu, J. Wang, J. Zhu, Y. Zhang, *J. Membr. Sci.* **2022**, *644*, 120074.

[39] P. Jin, S. Chergaoui, J. Zheng, A. Volodine, X. Zhang, Z. Liu, P. Luis, B. Van der Bruggen, *J. Hazard. Mater.* **2022**, *421*, 126716.

[40] R. Li, S. Cao, X. Feng, J. Don, X. Guo, H. Wang, Y. Zhang, *Sep. Purif. Technol.* **2022**, *300*, 121941.

[41] J. Zheng, R. Zhao, A. A. Uliana, Y. Liu, D. de Donnea, X. Zhang, D. Xu, Q. Gao, P. Jin, Y. Liu, A. Volodine, J. Zhu, B. Van der Bruggen, *Chem. Eng. J.* **2022**, *434*, 134705.

[42] Y. Wang, C. Bao, D. Li, J. Chen, X. Xu, S. Wen, Z. Guan, Q. Zhang, Y. Ding, Y. Xin, Y. Zou, *J. Membr. Sci.* **2022**, *661*, 120925.

[43] J. He, H. Wu, F. Ni, F. Shen, Y. Zhang, Z. Cheng, M. Huang, L. Zhao, L. Luo, Y. Zhang, X. An, *Sep. Purif. Technol.* **2023**, *318*, 123983.

[44] Q. Li, Z. Liao, X. Fang, J. Xie, L. Ni, D. Wang, J. Qi, X. Sun, L. Wang, J. Li, *Desalination* **2020**, *479*, 114343.

[45] R. Zhao, P. Jin, J. Zhu, Y. Li, G. Li, A. Volodine, Y. Liu, J. Zheng, B. Van der Bruggen, *J. Membr. Sci.* **2023**, *673*, 121477.

[46] L. Cheng, Y. Xie, X. Li, F. Liu, Y. Wang, J. Li, *J. Membr. Sci.* **2023**, *677*, 121632.

[47] Y. F. Mi, N. Wang, Q. Qi, B. Yu, X. D. Peng, Z. H. Cao, *Sep. Purif. Technol.* **2020**, *248*, 117079.
